# Syndrome “basses richesses” disease induced structural deformations and sectorial distribution of photoassimilates in sugar beet taproot revealed by combined MRI-PET imaging

**DOI:** 10.1016/j.plaphe.2025.100053

**Published:** 2025-05-15

**Authors:** Kwabena Agyei, Justus Detring, Ralf Metzner, Gregor Huber, Daniel Pflugfelder, Omid Eini, Mark Varrelmann, Anne-Katrin Mahlein, Robert Koller

**Affiliations:** a Institute for Bio- and Geosciences, Plant Sciences (IBG-2), Forschungszentrum Jülich GmbH, 52428, Jülich, Germany; b Institute of Sugar Beet Research (IfZ), Holtenser Landstraße 77, 37079, Göttingen, Germany

**Keywords:** *Beta vulgaris* (sugar beet), Syndrome “basses richesses”, magnetic resonance imaging, Positron emission tomography, Biotic interaction

## Abstract

The disease syndrome “basses richesses” (SBR) leads to a significant reduction in sugar beet biomass and sugar content, negatively affecting the sugar economy. The mechanistic understanding regarding growth and photoassimilates distribution within the sugar beet taproot diseased with SBR is currently incomplete. We combined two tomographic methods, magnetic resonance imaging (MRI) and positron emission tomography (PET) using ^11^C as tracer, to non-invasively determine SBR effects on structural growth and photoassimilates distribution within the developing taproot over six weeks. MRI analysis revealed a deformed cross-sectional anatomical structure from an early stage, as well as a reduction in taproot volume and width of inner cambium ring structures of up to 26 and 24 ​%, respectively. These SBR disease effects were also confirmed by post-harvest analysis of the taproot. PET analysis revealed a heterogeneous distribution of labeled photoassimilates for diseased plants: sectors of the taproot with characteristic SBR symptoms showed little to very low ^11^C tracer signal. The heterogeneity of SBR disease effects is most likely due to a partial inoculation of leaves leading to an uneven distribution of the SBR pathogen in the taproot through the strong vascular interconnection between shoot and root. Also, the pathogen needs to spread non-uniformly within the taproot to explain the observed marked increase of the SBR disease effects over time. Our results indicate that SBR affects photoassimilates sink capacity at an early stage of taproot development. Co-registration of MRI and PET may support an early judging of susceptibility and selection of promising genotype candidates for future breeding programs.

## 1 Introduction

Syndrome “basses richesses” (SBR) is a fast spreading sugar beet disease in mid Europe [1,2]. The cixiid planthopper *Pentastiridius leporinus* (L.) is the main vector of the proteobacterium ‘*Candidatus* Arsenophonus phytopathogenicus' and the phytoplasma ‘*Candidatus* Phytoplasma solani’ that cause SBR [[3], [4], [5]]. SBR symptoms appear in above and belowground organs of sugar beet. Aboveground symptoms include narrowing of new shoot organs and chlorosis of old leaves [3,6,7]. The most characteristic symptom in below-ground sugar beet organ is a brownish discoloration of the vascular tissues observed in slices of the taproot [6,7]. SBR leads to significant reductions of up to c. 29 ​% in taproot biomass [3] and a decrease in sugar content from, e.g., c. 18 to 13 ​% [4], thus negatively affecting the sugar economy. The vector and pathogens involved in SBR are already extending to other crops. Recent reports confirmed the presence of the disease in potato fields [8,9]. Moreover, the pathogen, ‘*Ca.* A. phytopathogenicus’ has been detected in onion samples [10]. Integrated management strategies, such as agronomic measures, breeding for tolerant or resistant varieties and plant protection are under investigation [11]. So far, promising approaches for agricultural practice are missing. The development of control strategies requires in-depth understanding of how SBR affects taproot morpho-physiological development [12]. It has been reported that the phloem sap content [12] and the phloem integrity [7] from source to sink elements of sugar beet are altered by SBR. But mechanistic understanding regarding photoassimilates transport and accumulation in taproots diseased with the SBR pathogens is currently incomplete.

The plant vascular architecture plays a major role for the transport of photoassimilates from shoots to roots [13,14]. The sugar beet plant presents a complex anastomosis where specific leaves attach themselves to sections of cambium rings [15]. These cambium rings are composed of a vascular ring and are approximately equidistant from one another. In between the vascular rings are broad bands of storage parenchyma where the sugar beet stores most of its sugars [15]. Transmission of SBR causing proteobacteria is restricted to the phloem [7]. The activity of the proteobacteria may lead to the occlusion of sieve-tube elements, impairing photoassimilate transport in the phloem. Similar interactions have been reported by Musetti et al. [16] for the *Ca*. Phytoplasma vitis and *Vitis vinifera* pathosystem. To our knowledge detailed studies of photoassimilate transport and distribution within the taproot during SBR-sugar beet interactions are missing and may reveal relevant insights on how SBR affects the sink organ of the growing plant. Recent studies regarding the SBR disease focused on the alteration in the chemical composition of the phloem of diseased plants, molecular detection of the pathogens and molecular characterization of the transmission vector, mass rearing of the vector, its alternate host and probable agronomic practices to reduce to the vector population [1,2,[8], [9], [10], [11],[17], [18], [19], [20]].

Employing sensor-based phenotyping technologies offers non-invasive approaches for elucidating morpho-physiological mechanisms that link pathogen infection and dynamics of disease symptoms in host plants [21]. These may allow an early detection and characterization of disease-related changes in plant growth. Progress towards utilizing sensor-based technologies to quantify the damage caused by pathogens in sugar beet has mostly been achieved for above-ground traits using optical sensors [[22], [23], [24], [25], [26]]. Due to the opaque nature of soils, detailed knowledge regarding disease occurrence and symptom progression on belowground taproot is limited, even though the taproot forms the main economic value of sugar beet. This gap could be closed by the application of tomographic technologies like magnetic resonance imaging (MRI) [[27], [28], [29], [30], [31]] and positron emission tomography (PET) [27,32,33]. MRI has been employed to study the detailed anatomical features of the sugar beet taproot [29]. For sugar beet-biotic interactions, MRI was used to detect taproot anatomical alterations caused by *Cercospora* leaf spot [30]. Also, the damage caused by *Heterodera schachtii* and *Rhizoctonia solani* on sugar beet taproot was detected by MRI [28]. After supplying ^11^CO_2_ to plant leaves, PET allows for non-invasive 3D detection of the ^11^C tracer and assessment of distribution of recently fixed photoassimilates in plant organs [27,[32], [33], [34], [35], [36]]. Combination of PET with other imaging technologies like MRI or X-ray computed tomography was used for characterizing the dynamics in translocation of photoassimilates within belowground organs of different plant species [27,32,35,36]. These studies indicate the potential of multimodal imaging for investigating structural and functional effects exerted by pathogens on belowground crop organs. However, changes in carbon distribution in sugar beet taproot due to pathogen infection have not been in studied *in vivo* so far.

In our present study, we employed MRI and PET to uncover tempo-spatial effects of SBR on belowground taproot development. We observed non-invasively the effects of the phloem-restricted pathogen causing SBR, ‘*Ca.* A. phytopathogenicus’, on cross-sectional taproot features and development of taproot volume over several weeks, complemented by the investigation of temporal and spatial photoassimilates distribution within the taproot. With this approach we wanted to answer the following research questions: from which time point in sugar beet development do SBR symptoms become visible in the taproot? How does severity of SBR symptoms in the taproot progress over time? Assuming that SBR symptoms are not evenly distributed over the taproot volume: does this distribution change over time?

## 2 Materials and methods

### 2.1 Experimental design

**Fig. 1:**
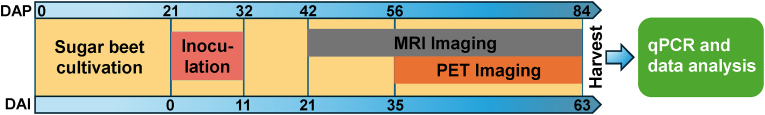
Schematic of experimental timeline and processes. Key steps include plant cultivation, SBR disease transmission (inoculation), MRI-PET imaging phase and qPCR-based discrimination between diseased and control samples after harvest. DAP ​= ​days after planting, DAI ​= ​days after inoculation.

**Fig. 2:**
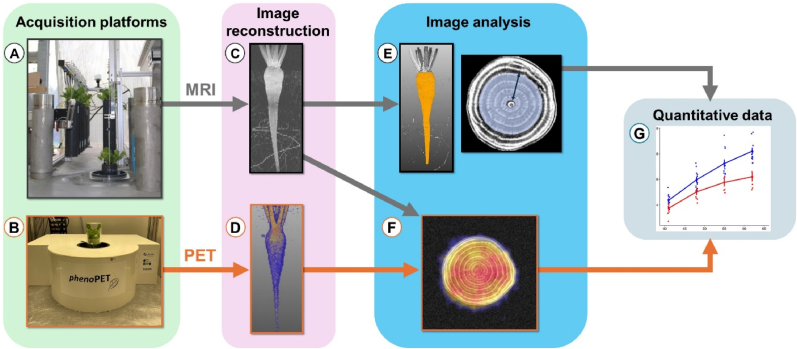
Workflow of image data acquisition and analysis. (A) Magnetic resonance imaging (MRI) acquisition platform; (B) Positron emission tomography (PET) acquisition platform; (C) MRI data reconstruction; (D) PET data reconstruction; (E) Taproot segmentation (determined taproot volume in orange) and cambium ring contouring (marked by blue circle with arrow); (F) MRI-PET co-registration, grey image parts represent MRI, colored image parts represent PET; (G) Quantitative trait analysis and visualization.

Multiple plants of sugar beet (*Beta vulgaris* L.) were grown under controlled conditions and subjected to two treatments, non-inoculated and inoculated with the SBR pathogen ‘*Ca*. A. phytopathogenicus’. Two non-invasive tomographic imaging techniques (MRI and PET) were employed for belowground taproot phenotyping. All plant samples were subjected to MRI measurements after the inoculation access period. For PET measurements, a subset of samples from each treatment was selected randomly. MRI and PET acquisitions were performed over a period of six weeks to follow growth dynamics and disease progression. The timeline of the experiments is depicted in Fig. 1. The acquired MRI and PET images were reconstructed followed by taproot traits analysis and further quantification of disease effects (Fig. 2).

### 2.2 Soil substrate preparation

MRI compatible soil substrate (Speyer 2.1, LUFA Speyer, Germany, characterized in Pflugfelder et al. [37]) was oven-dried at 60 ​°C for 24 ​h, demagnetized and prewetted. The resulting soil was filled into PVC pots of 400 ​mm height and 81 ​mm inner diameter covered with a nylon meshed-perforated bottom. Soil moisture was maintained at c. 20 ​% volumetric water content by regularly watering pots to keep pot weight at a set value established initially with dried soil.

### 2.3 Plant cultivation

A single seed of sugar beet (*Beta vulgaris* L.) genotype BTS 8750 (uncoated) (Betaseed GmbH, Frankfurt, Germany) was planted into each pot at depth of 1 ​cm. Plants were grown in a climate chamber at 24 ​°C: 16 ​°C temperatures, 16 ​h: 8 ​h light: dark, and 60 ​% relative humidity. Light was sourced from LED panels (CreeLED Inc, Shanghai, China) with an intensity of 680 ​μmol ​m^−2^ ​s^−1^ at plant level.

### 2.4 SBR transmission assay

For the transmission assay, 21 days old sugar beet plants were transported from Forschungszentrum Jülich (FZJ) to Institute of Sugar beet Research (IfZ) and kept at the planthopper rearing facility of the IfZ. *P*. *leporinus* adults diseased with γ-3 proteobacterium ‘*Ca*. A. phytopathogenicus’ were acquired from the planthopper rearing facility of IfZ according to Pfitzer et al. [11]. Per tent, four healthy sugar beet plants (BBCH stage 11–12) were exposed to 20 ​*P. leporinus* planthoppers in 60∗60∗60 ​cm rearing tents with a mesh size of 150 ​μm (BugDorm-2120F, Insect rearing Tent, MegaView Science Co., Ltd., Taichung, Taiwan) for an inoculation access period of 11 days. For the negative control group, plants were kept in rearing tents without planthoppers. During this period, plants and planthoppers were kept under controlled conditions at 22.77 ​± ​0.98 ​°C, 43.39 ​% ​± ​6.47 ​% relative humidity and 16h: 8h, light: dark. Light was sourced from a full-spectrum LED panel (Valoya, RX 400, Spektrum NS 1, Helsinki, Finland). The light intensity within the tent was set at 250 ​μmol ​(s ​m^2^)^−1^. At the end of the inoculation access period, all plants were sprayed with the insecticide Imidacloprid (Bayer AG, Frankfurt, Germany) at a concentration of 0.035 ​% according to manufacturer's manual to eradicate the planthoppers. Plants were inspected three days after spraying to ensure that all planthoppers were eliminated prior to the return of the plants to FZJ. At FZJ, plants were cultivated at standard conditions as stated in the plant cultivation section above. We set the time when plant hoppers were introduced in the tents as reference time for the definition of days after inoculation (DAI). Since the measurements of each sample run had to be performed on more than one day, there were time differences between the single samples of up to 13 ​h for MRI and up to 30 ​h for PET. As a consequence, measurement times expressed as DAI have an uncertainty of approximately 1 day. In order to prevent a bias in the results we randomly changed the order of plants at each PET imaging date.

### 2.5 MRI measurements

Belowground sugar beet organs were imaged weekly using MRI [31]. The MRI set-up includes a robot system which enables automated image acquisition (Fig. 2A). The MRI consists of a 4.7 ​T magnet (Magnex, Oxford, UK) equipped with a MRS console (MR Solutions, Guildford, UK). The vertical orientation of the magnet enables acquiring plant images in their natural vertical inclination. A radio-frequency coil with an inner diameter of 100 ​mm (Varian, Palo Alto, CA, USA) was used. MR images were acquired with a Spin-Echo Multi-Slice sequence with the following parameters: Repetition time ​= ​1250 ​ms, bandwidth ​= ​400 ​kHz, horizontal slices with 2.0 ​mm thickness, in plane resolution 0.2∗0.2 ​mm^2^, matrix size 500∗500∗50, echo time ​= ​10 ​ms, two averages. The measurement time was approximately 21 ​min for a soil volume of 10∗10∗10 ​cm^3^.

### 2.6 ^11^CO_2_ tracer production, gas exchange system and labelling approach

^11^CO_2_ tracer was produced onsite at an 18 ​MeV fixed-energy cyclotron (IBA Molecular Europe, Louvain-la-Neuve, Belgium). For each shoot labelling, the ^11^CO_2_ from the cyclotron was trapped on a molecular sieve [38]. The trapped ^11^CO_2_ was recovered by heating it up to 200 ​°C and subsequently flushed into the administration cycle of the gas exchange system. Sugar beet plants were mounted in the field of view of the plant dedicated PET system “*pheno*PET” [33]. The whole shoot of sugar beet plant was enclosed in a ^11^CO_2_ labelling cuvette. Different cuvette sizes of 170, 210 or 260 mm height with a diameter of 81 ​mm were employed depending on plant age and height. The cuvette was air-tightened and connected to the gas exchange and the ^11^CO_2_ application system. The serial connections between the gas exchange system, the ^11^CO_2_ tracer application system and the cuvette enabled gas exchange measurements and the parallel administration of ^11^CO_2_ to the whole shoot of the sugar beet plants. Each sequence of ^11^CO_2_ administration was done with approximately 50 MBq of ^11^CO_2_ for a period of 6 ​min. Details about functionality of the gas exchange system and procedures of releasing ^11^CO_2_ in the cuvette were described in Metzner et al. [32].

### 2.7 PET image acquisition

*pheno*PET was used to acquire tomographic images of ^11^C tracer within the taproot (Fig. 2B). The bore of the *pheno*PET is built in a vertical orientation and has a cylindrical field of view of 180 ​mm in diameter and 202 ​mm in height. The resolution of acquired tomographic images comprises of voxel size of 0.9 ​× ​0.9 ​× ​1 ​mm with spatial resolution of 1.8 ​mm. *pheno*PET is installed in a climate chamber to provide optimal climate conditions for plants during measurements. The climate conditions in this chamber were similar to the plant cultivation climate chamber. Prior to PET measurements, plants were allowed to acclimate until CO_2_ assimilation was stabilized. Image data was acquired for 150 ​min after each ^11^CO_2_ pulse labelling. After each measurement, the plants remained in the *pheno*PET climate chamber until the next day and afterwards were transferred back to the cultivation climate chamber.

### 2.8 PET image reconstruction

PET images were reconstructed into 30 frames with 5 ​min duration [33]. Scatter and attenuation corrections were not employed. The individual frames were decay corrected such that the image intensity was proportional to the tracer amount in each frame. For data analysis and visualization, we used a maximum intensity projection of the PET tracer over time (Fig. 2D).

### 2.9 MRI and PET image analysis

After MRI and PET image acquisitions MeVisLab software (version 3.6.1, MeVis Medical Solutions AG, Bremen, Germany) was used to visualize and analyze structural features as well as dynamic ^11^C tracer distribution patterns inside the taproot.

### 2.10 Analysis of MRI images of taproot

For taproot volume quantification, a binary image was generated from MR images (Fig. 2C), using an image intensity threshold. Fine structures such as root segments were removed using a median filter and subsequently selecting the largest connected component. Finally, the leaf base was selected manually. The remaining volume defined the taproot (Fig. 2E). For taproot cross-sectional visualization and quantification, MRI data was visualized in 2D. A qualitative as well as quantitative analysis of taproot cross sectional features was done based on 2D slices at three different vertical positions of the taproot. The positions were chosen similar to the cross-sectional slices at final harvest (Supplementary Fig. S1). For quantitative analysis, the ring structures of each taproot sample were contoured manually while excluding the central core (Fig. 2E). The mean distance between adjacent contours represented the ring width. Innermost ring width was calculated by summing the width of rings 1–4 (Fig. 2E).

### 2.11 MRI-PET co-registration

To gain insights into ^11^C distribution patterns, MRI and PET images were co-registered manually. For image display (Fig. 2F) we used a fixed color scale for all PET images. For MRI images, the color scale needed to be adapted manually between the different time points to compensate for signal changes due to different tuning and matching settings necessary to accommodate the growing taproot and different soil water levels. To distinguish both modalities, MRI images were presented in grey values whilst PET images were shown in color (Fig. 2F).

### 2.12 Determination of intra taproot tracer distribution heterogeneity

The tracer distribution was analyzed in 10 slices of 5 ​mm thickness each, spread over the taproot. Tracer heterogeneity (*H*) was defined as *H* = (*p*_80_ – *p*_20_)/*p*_50_, with *p*_N_ being the N-th percentile of the tracer distribution. Mean heterogeneity and standard deviation were estimated from heterogeneity of all 10 slices.

### 2.13 Post harvest taproot biomass estimation

Taproots were excavated from pots and cleaned from soil by washing at 63 DAI directly after the last MRI and PET measurements. Fine roots were removed from the taproot with a scalpel. The cleaned taproots were wrapped in paper towels to absorb excess water. Subsequently, they were unwrapped, and their fresh biomass was obtained by weighing on a laboratory balance (Mettler-Toledo GmbH, Giessen, Germany). The taproot diameter was measured with a caliper at the thickest part of the taproot.

### 2.14 Taproot tissue sampling and qPCR detection of ‘*Ca.* A. Phytopathogenicus’

Taproot tissue samples were collected from all plants after harvest at three different cross-sections of the taproot (upper section: directly below the pith, mid-section: 2 ​cm below upper portion and lower section: 3 ​cm below mid-section) (Supplementary Fig. S1). Taproot tissue samples were collected in 1.5 ​ml centrifuge tubes (Eppendorf AG, Hamburg, Germany) and stored in −80 ​°C until further analysis. Nucleic acid extraction to determine infection by ‘*Ca*. A. phytopathogenicus’ was performed using the MagMAX Plant DNA kit (Thermo Fisher Scientific, Frankfurt, Germany) for all inoculated samples and two non-inoculated samples. The absolute DNA concentration was estimated by using Nanodrop (Ds-11 Spectrometer, Denovix, Wilmington, USA) and diluted with sterile water to a final concentration of c. 20 ​ng ​μl^−1^. Samples were later transferred into 96-well plates (Bio-Rad Laboratories GmbH, Feldkirchen, Germany) for further analysis.

Primer sequences (Supplementary Table S1) as designed and described in Zübert and Kube [39] were used to target HSP20 gene sequences of ‘*Ca*. A. phytopathogenicus’. A qPCR reaction mix comprised 6.3 ​μl distilled water, 10 ​μl Maxima Probe qPCR Mix, 0.9 ​μl of each primer, 0.4 ​μl of Probe (10 ​μM; Fam-BHQ1 labeled) and 1.5 ​μl of DNA template. PCR was conducted on a CFX96 real time system C1000 touch thermal cycler (Bio-Rad, Feldkirchen, Germany). qPCR conditions were 95 ​°C for 3 ​min, 40 cycles at 95 ​°C for 15 ​s, 60 ​°C for 30 ​s. The resulting data was analyzed via Bio-Rad CFX Manager software (version 3.1). In this design, samples can be considered diseased if Cq values are ≤35.00 or non-diseased if Cq values are >35.00.

### 2.15 Statistical analysis

*R* statistical computing software (version 4.2.1: packages; ggplot2, tidyverse, rstatix and ggpubr) was used for analysis of the data for taproot volume, inner rings and intra taproot tracer heterogeneity. Differences among means were tested using a *t*-test. Significance levels were set at ∗∗∗p ​< ​0.001, ∗∗p ​< ​0.01 and ∗ p ​< ​0.05.

## 3 Results

### 3.1 Identification of diseased taproot samples by qPCR analysis and detection of SBR symptoms after harvest

**Fig. 3:**
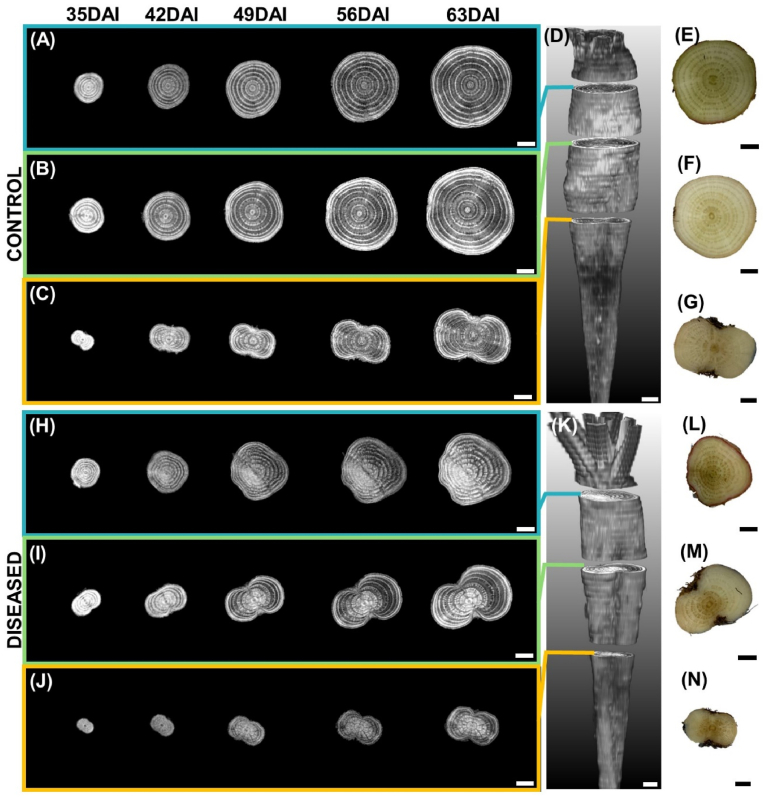
Examples of cross-sectional anatomical features at three vertical positions of control taproot C1 (A–G) and diseased taproot D1 (H–N). Grey images (A, B, C, H, I, J) represent slices of MRI acquired at five time-points after inoculation (35, 42, 49, 56 and 63 DAI). (D, K) indicate vertical positions where slices were obtained for control and diseased taproot, respectively. (E, F, G, L, M, N) RGB images show a cross-section of the same control (E, F, G) and diseased (L, M, N) taproots after destructive harvest at 63 DAI. Brownish discoloration in (L, M, N) shows SBR symptoms. Bars, 0.5 ​cm.

Our experimental set-up consisted of 10 inoculated and 10 non-inoculated sugar beet plants. Based on qPCR analysis, we confirmed the presence of ‘*Ca.* A. phytopathogenicus’ in 8 out of the 10 inoculated plants. Cq values for diseased samples ranged between 23.6 and 29.5 and ‘*Ca.* A. phytopathogenicus’ was detected in three sections in each diseased plant (Supplementary Table S2). In addition, cross sections of all diseased taproot showed brownish discoloration of the vascular bundles at harvest (Fig. 3L, M, N, and Supplementary Fig. S13–S19). Brownish discolorations were localized to specific regions and in some cases were surrounded by healthy tissues (Fig. 3, and Supplementary Fig. S13–S19). Brownish discoloration was not observed in taproot samples of non-inoculated plants and plants that showed negative for the proteobacterium by qPCR (Fig. 3E, F, G, and Supplementary Fig. S2–S12). Based on the results of the qPCR analysis and the symptoms observed during destructive analysis, we defined control as non-diseased plants (10 non-inoculated plus two non-diseased inoculated samples). In the following, we number control samples as C1-12 and diseased samples as D1-8.

### 3.2 SBR effects on taproot diameter and fresh weight after harvest

At harvest the infection with the SBR proteobacterium reduced fresh weight of the taproots on average from 43.6 ​± ​1.3 ​g of control to 33.6 ​± ​2.1 ​g (mean ​± ​SE) of diseased taproots. Similarly, mean taproot diameter at harvest was reduced from 29.6 ​± ​0.5 ​mm for control plants to 25.7 ​± ​0.8 ​mm (mean ​± ​SE) for diseased taproots (Supplementary Table S3).

### 3.3 SBR effects on taproot development

**Fig. 4:**
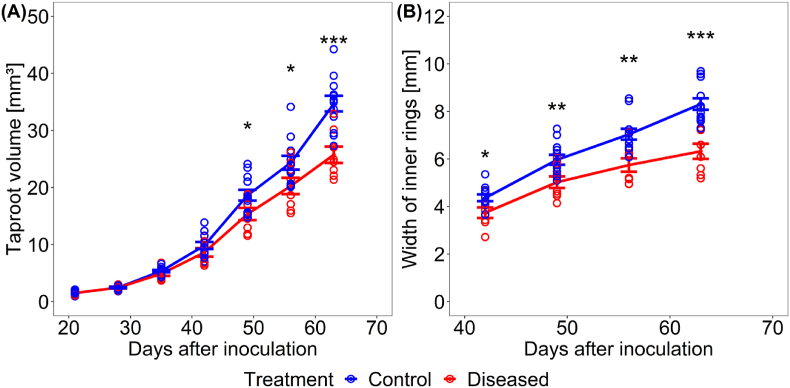
Quantification of taproot development by MRI. (A) Taproot volume and (B) inner ring width of control (blue) and diseased (red) taproots over time. Circles represent single values and lines represent their means. Number of replicates n ​= ​12 for control at 21, 28, 35, 49 and 63 days after inoculation (DAI); n ​= ​11 for control at 42 and 56 DAI; n ​= ​8 for diseased at 21, 28, 39, 56 and 63 DAI; n ​= ​7 for diseased at 35 and 42 DAI. Error bars represent standard error of mean values. Asterisk marks indicate significant differences in a *t*-test, ∗∗∗p ​< ​0.001, ∗∗p ​< ​0.01, ∗p ​< ​0.05.

To analyze SBR effects on structural taproot development, MRI of belowground taproot was imaged weekly from 21 DAI until 63 DAI. Temporal MRI acquisition was achievable for all samples, except for measuring dates 35, 42 and 56 DAI where one, two and one measurements were unsuccessful, respectively, due to technical problems. Apart from this unsuccessful imaging, the mean of diseased plants for taproot volume and inner rings represented 8 samples whilst mean of control plants represented 12 samples. Quantitative image data analysis revealed a significant, progressing reduction in taproot volume in the presence of the SBR proteobacterium. Reduction in taproot volume was significant at 49 DAI until 63 DAI (Fig. 4A). Diseased samples showed a 12, 18, 17 and 26 ​% reduction relative to control samples at 42, 48, 55 and 63 DAI, respectively. Analysis of temporal development of inner taproot ring width showed a significant reduction by the presence of the SBR proteobacterium. Reduction in inner rings was significant earliest at 42 DAI until 63 DAI (Fig. 4B). Diseased samples showed a 16, 17, 19 and 24 ​% decrease relative to control samples at 42, 48, 55 and 63 DAI, respectively.

### 3.4 SBR effects on taproot cross-sectional anatomical features

**Fig. 5:**
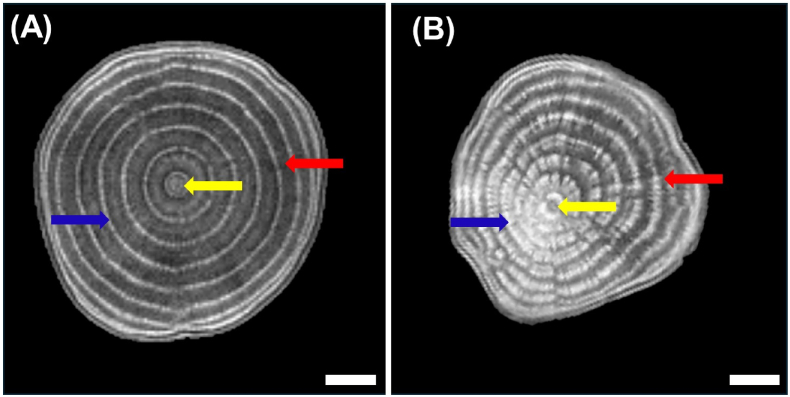
Comparison of MRI signal intensity and cross-sectional structures of (A) control taproot C1 and (B) diseased taproot D1 at 63 days after inoculation. Yellow arrows indicate central core, red arrows indicate storage parenchyma and blue arrows indicate cambium ring structure. Diseased taproot shows brighter and smeared ring structure with less phloem parenchyma area as compared to control taproot. Bars, 0.5 ​cm.

From 2D slice images of taproot obtained from MRI, we could differentiate cambium rings, central core, and storage parenchyma for both control and diseased plants (Fig. 3, Fig. 5). The MRI signal of diseased tissue was clearly distinguishable from healthy tissue in diseased plants. This was prominent for all of the three cross-sections at different vertical positions of the taproot (Fig. 3H, I, and J). MRI of diseased taproot showed deformed cambium rings with broader indefinite patterns unlike in diseased taproot where cambium structures were definite (Fig. 5). Moreover, sections of cambium rings showed a brighter image signal. The pattern of deformations observed by MRI was similar to the brownish colored portions of the taproot cross-sections observed during destructive analysis at harvest.

### 3.5 SBR effects on distribution of recently fixed photoassimilates within taproot

The effects of SBR on distribution of recently fixed photoassimilates was determined by co-registration of PET images with the structural MRI images (MRI-PET). PET acquisition was performed for three non-inoculated and three inoculated samples, except that one image was unsuccessful during acquisition at imaging day 42 DAI. Two out of the three inoculated plants dedicated for PET acquisition tested positive for ‘*Ca.* A. phytopathogenicus’. Therefore, in the PET and subsequent heterogeneity analysis, the mean of control plants represented four samples whilst the mean of diseased plants represented two samples apart from the reported unsuccessful imaging.

**Fig. 6:**
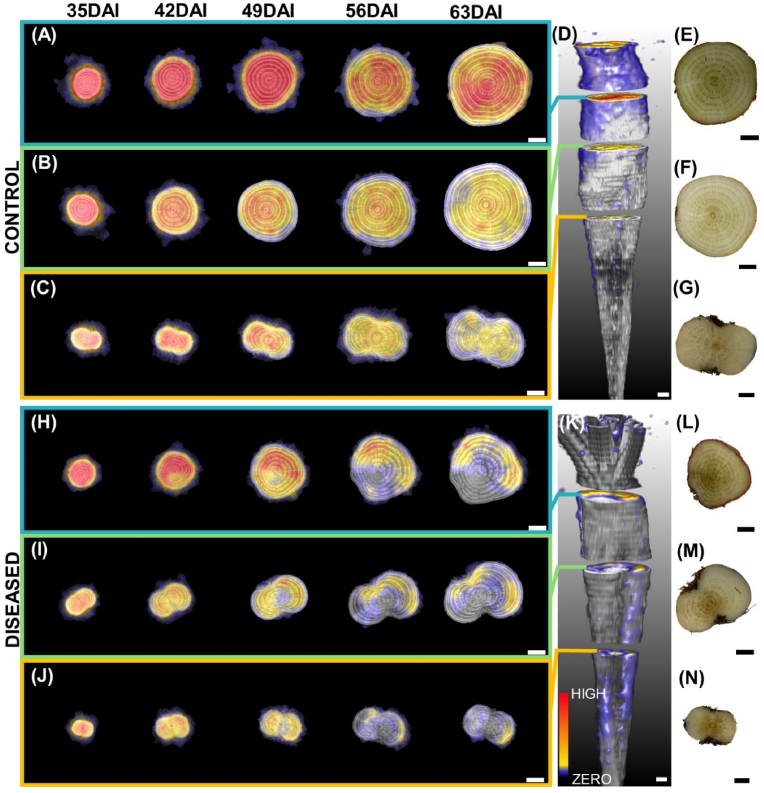
Examples of MRI-PET co-registration showing tracer distribution in the developing taproot as well as RGB images of taproot at destructive analysis for control taproot C1 (A–G) and diseased taproot D1 (H–N). Figure layout is same as in Fig. 4 except for additional PET images overlaid in color. Areas with cooler colors (grey, blue) indicate no or very low ^11^C-tracer activity while areas with warmer colors (yellow and red) indicate high ^11^C- tracer activity. Bars, 0.5 ​cm.

**Fig. 7:**
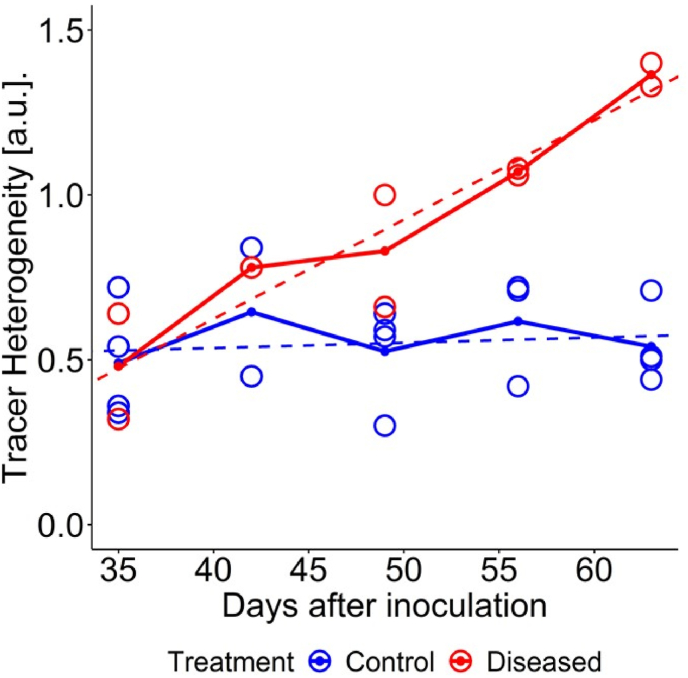
Temporal development of mean heterogeneity *H* of tracer distribution within taproots. Heterogeneity was averaged over 10 slices of taproot for control (blue) and diseased (red) taproots, respectively. Circles represent values per taproot and lines represent their means. Blue and red dotted lines represent linear regressions for control (R^2^ ​= ​0.011) and diseased (R^2^ ​= ​0.859) taproots, respectively. Number of replicates n ​= ​4 for control at 35, 49 and 63 DAI; n ​= ​2 for control at 42 DAI; n ​= ​3 for control at 56 DAI; n ​= ​2 for diseased at 35, 49, 56 and 63; n ​= ​1 for diseased at 42 DAI. Individual intra taproot mean and standard deviation values for slices are displayed in Supplementary Table S4.

For qualitative analysis, we considered 2D slices at three different vertical positions of the taproot that were similar to the cuttings for determining the presence of SBR protobacterium by qPCR at final harvest. For control samples, photoassimilates were distributed homogenously over the taproot slices (Fig. 6A, B, C, Supplementary Fig. S20–S22 and Video S1). In the presence of the SBR proteobacterium we found a sectorial distribution of recently fixed photoassimilates (Fig. 6H, I, J, Supplementary Fig. S23 and Video S2). The signal intensity of tracer was either missing or very low for a sector of the developing taproot. The sectorial distribution was visible from 42 DAI and was predominant at later imaging dates (56 and 63 DAI). This sectorality was extending throughout the taproot from top to bottom (Video S2). Sectors of the taproot with low to no tracer signal were similar to areas that showed brownish discoloration. For quantitative analysis we determined heterogeneity *H* of tracer distribution within 10 slices of the taproot (Fig. 7, and Supplementary Table S4). Heterogeneity *H* increased by a factor of 2.8 in diseased plants over the course of the experiment. In control plants, the tracer distribution within the taproot remained homogeneous (Fig. 7, and Supplementary Table S4). In general, the intensity of tracer signal in the developing taproot detected by PET was reduced for all plants. This could be explained by the fact that the same amount of ^11^C tracer was being diluted into larger taproot volume at later imaging periods.

## 4 Discussion

We employed MRI and PET to uncover temporal-spatial effects of the biotic stressor SBR on belowground taproot development. To our knowledge, this was the first time that individual plant development was monitored non-invasively with MRI-PET over such a long time period. Also, this study was the first to analyze the effect of a biotic stressor on photoassimilates distribution within individual sugar beet taproots.

### 4.1 SBR disease effects on morphological and physiological sugar beet taproot development

Repeated MRI measurements showed a decrease in volume and inner ring width of diseased taproot relative to control taproot samples. A similar result was reported by Schmittgen et al. [30] for sugar beet diseased with *Cercospora* leaf spot. The symptoms observed by MRI started at 42 DAI and continued progressively until harvest at 63 DAI, when plants were 84 days old (Fig. 3). While the effect of SBR pathogen on taproot volume did not start before 49 DAI, cambium ring structure could have been affected even earlier. But cambium ring analysis was possible earliest at 42 DAI due to the time required for secondary thickening of the taproot. At this stage, the four innermost rings were clearly defined and separated from each other by phloem parenchyma unlike outer ring structures which could not be distinguished (Fig. 3). This was expected, since the widening of phloem parenchyma due to storage is observed in the innermost rings of the taproot at this stage [15].

In addition to inner ring width, we detected qualitative changes in vascular structures exerted by SBR (Fig. 3, Fig. 5), also as early as 42 DAI. Deformations of parts of the vascular structures are some of the microscopic and macroscopic symptoms known to be present in the taproot of SBR diseased plants [7]. However, there is no clear explanation yet for the observed broadened and interrupted cambium rings in cross-sections of the taproot (Fig. 5B). These features were confined to specific sectors of the observed cross-sections and could be distinguished from surrounding tissues which were looking healthy. Affected regions identified early with MRI and brownish discolored regions seen in the destructive analysis conformed with the sectors of the taproot which received little to no recently fixed photoassimilates (Fig. 6H, I, and J). The latter could be detected from DAI 49 onwards, i.e., one week later than the symptoms detected with MRI, probably because of the lower spatial resolution of PET compared to MRI.

### 4.2 Linking shoot architecture to distribution of belowground SBR disease symptoms

The inoculation period started at BBCH stage 11–12 and ended at BBCH stage 14, i.e., when there were just two to four fully expanded leaves. These leaves initiate the formation of the three innermost cambium rings and later stay connected to maintain an intimate relationship between leaves and cambium rings [15]. Since phloem-restricted pathogens are translocated with photoassimilates from source to sink organs [40,41], the pathogen infection can be assumed to be established in the innermost cambium. Development of outer rings after the inoculation period was not directly affected by the pathogen, explaining the observation of infected inner rings surrounded by healthy tissue (Supplementary Fig. S17, and S19).

The specific sectorality of disease symptoms we observed in some cases (Fig. 3H, L, and Fig. 6H, L) could be explained by a possible selective nature of the transmitting vector. Selection of a specific side of the shoot architecture or leaf by the vector will be directly proportional to abundance of SBR pathogens at a specific sector of the taproot, because vascular architecture plays a relevant role in the phloem sap distribution [27,42].

### 4.3 Possible mechanistic explanations for observed SBR disease symptoms

The relative difference in taproot volume and inner ring width increased over time for control and diseased plants (Fig. 4). In parallel, the heterogeneity of tracer distribution increased over time (Fig. 7). This indicates that SBR effects on taproot physiology and growth worsened as the disease progressed, which would be possible only if the pathogen spread inside the taproot. We suggest that the increasing portions of the cross-section with little to no signal in the PET measurements (Fig. 6H–J) are related to a growing abundance of the pathogen in these regions.

The observed distribution patterns of photoassimilates could be due to low metabolic activity of adjoining leaves which supplied photoassimilates to specific taproot sections. Schmittgen et al. [30] suggested this mechanism for their observation of reduced growth of inner ring structures under pathogen attack, albeit for a different pathosystem. Another explanation would be that the distribution pattern is driven by reduced metabolic activity of both source and sink cells. In a case of a source leaf with little or no metabolic activity, neighboring source leaves might compensate for the supply of photoassimilates to other parts of the sink. This would be possible due to the subtle existence of vascular connections between leaves and cambium rings. Such compensatory mechanism in resource distribution have been observed in a case of partially defoliated sugar beet [42]. Owing to this and the observed abnormalities in MRI as well as brownish discolored tissue in destructive analysis, we are suggesting that the observed sectorality is driven by dead sink cells, which leads to a compromised structural integrity exerted by SBR. Thus, SBR weakens host tissue structure and may cause leakage of cellular contents. The leakage could be the reason for the abnormal cambium ring formation observed in MRI. Another explanation could be that the SBR pathogen might secrete effectors or toxins in the phloem (as suggested by Christensen [40] for a different phloem-restricted pathogen) which trigger host responses and cause the morphological changes [43].

### 4.4 Perspectives for future studies

Our current approach enabled monitoring allocation dynamics of recently fixed photoassimilates, thus uncovering short term dynamics of tracer distributions. On the other hand, using ^11^C as tracer limits clues regarding relatively long-term remobilization of photoassimilates in the taproot. Long-term analysis of storage dynamics of photoassimilates may reveal how a stressor induces a switch in sink-source identities of the developing taproot [44]. Other carbon isotopes such as the stable ^13^C tracer [45] or long-lived ^14^C [42,44] could be used in combination with ^11^C to investigate short- and long-term carbon dynamics at the same time.

Apart from sizes, quality in the form of taproot shape [46] and tissue strength [47] are important parameters for sugar beet processing. Effects exerted on sugar beet tissue structure and content by pathogens affect the processing quality of taproot [48]. We assume that SBR will not only affect taproot volume as seen in our study, but also the geometry of the developing taproot. Therefore, further experimentation and implementation of algorithms for the detection and quantification of SBR effects on taproot geometry will be beneficial. Also, a detailed tissue characterization of taproot under specific stress scenarios would provide a strong basis for quantifying performance [49] and harnessing tissue strength in future breeding programs [47,50].

Further experimentation is needed to link shoot physiological traits to belowground taproot traits. This will provide a holistic overview of functional and structural relationships among above-and below-ground organs of sugar beet during SBR disease progression.

It might be possible to determine the distribution of the pathogen by using MRI-PET for image guided tissue sampling, followed by qPCR to quantify pathogen presence in contrasting regions of interest. The same approach of image guided-sampling with MRI-PET could also be applied to investigate the role of sucrose transporter genes during SBR pathogenesis. These may unravel molecular mechanisms regarding sucrose export out of source tissues to receiving sink tissues and may present novel opportunities towards improving crop performance [[51], [52], [53]].

## 5 Conclusion

This study is the first to non-invasively characterize SBR disease effects on sugar beet taproot by tomographic imaging. We observed sectorial distribution of recently fixed photoassimilates within the taproot, altered cross-sectional tissue organization and reduction in taproot development during SBR-sugar beet interaction. Further, we linked within taproot sector-specific symptoms detected by MRI-PET to observed SBR symptoms in the taproot during destructive analysis. Thus, our approach enabled an early detection and quantification of SBR induced damage on below-ground taproot of intact plants from which we could derive new insights on the progression of the pathogen within the host. This detailed characterization may be used as basis for judging susceptibility and selection of promising genotype candidates for future breeding programs.

## Author contributions

KA, JD, AKM, and RK: Conceptualization; KA, RM, GH, DP, OE, and MV: Investigation; RM, DP, MV, and AKM: Resources; DP: Software; KA: Formal Analysis; KA: Visualization; KA: Writing – Original Draft; KA, JD, GH, DP, OE, AKM, RK: Writing – Review & Editing; GH, AKM, and RK: Supervision; RK and AKM: Funding Acquisition.

## Data availability

All relevant data supporting the findings of this study are available in the main article or the Supplementary Data.

## Declaration of competing interest

The authors declare that they have no known competing financial interests or personal relationships that could have appeared to influence the work reported in this paper.

## References

[1] Pfitzer R., Rostás M., Häußermann P., Häuser T., Rinklef A., Detring J. (2024). Effects of succession crops and soil tillage on suppressing the syndrome 'basses richesses' vector Pentastiridius leporinus in sugar beet. Pest Manag. Sci..

[2] Duduk B., Stepanović J., Fránová J., Zwolińska A., Rekanović E., Stepanović M. (2024). Geographical variations, prevalence, and molecular dynamics of fastidious phloem-limited pathogens infecting sugar beet across Central Europe. PLoS One.

[3] Bressan A., Sémétey O., Nusillard B., Clair D., Boudon-Padieu E. (2008). Insect vectors (Hemiptera: cixiidae) and pathogens associated with the disease syndrome “basses richesses” of sugar beet in France. Plant Dis..

[4] Sémétey O., Bressan A., Richard-Molard M., Boudon-Padieu E. (2007). Monitoring of proteobacteria and phytoplasma in sugar beets naturally or experimentally affected by the disease syndrome ‘Basses richesses’. Eur. J. Plant Pathol..

[5] Bressan A., Terlizzi F., Credi R. (2012). Independent origins of vectored plant pathogenic bacteria from arthropod-associated Arsenophonus endosymbionts. Microb. Ecol..

[6] Mahillon M., Groux R., Bussereau F., Brodard J., Debonneville C., Demal S. (2022). Virus yellows and syndrome "basses richesses" in western Switzerland: a dramatic 2020 season calls for urgent control measures. Pathogens.

[7] Gatineau F., Jacob N., Vautrin S., Larrue J., Lherminier J., Richard-Molard M., Boudon-Padieu E. (2002). Association with the syndrome “basses richesses” of sugar beet of a phytoplasma and a bacterium-like organism transmitted by a Pentastiridius sp. Phytopathology.

[8] Behrmann S.C., Rinklef A., Lang C., Vilcinskas A., Lee K.-Z. (2023). Potato (Solanum tuberosum) as a new host for Pentastiridius leporinus (Hemiptera: cixiidae) and candidatus Arsenophonus phytopathogenicus. Insects.

[9] Rinklef A., Behrmann S.C., Löffler D., Erner J., Meyer M.V., Lang C. (2024). Prevalence in potato of 'candidatus Arsenophonus phytopathogenicus' and 'candidatus phytoplasma solani' and their transmission via adult Pentastiridius leporinus. Insects.

[10] Therhaag E., Ulrich R., Gross J., Schneider B. (2024). Onion (Allium cepa L.) as a new host for 'Candidatus Arsenophonus phytopathogenicus' in Germany. Plant Dis..

[11] Pfitzer R., Varrelmann M., Schrameyer K., Rostás M. (2022). Life history traits and a method for continuous mass rearing of the planthopper Pentastiridius leporinus, a vector of the causal agent of syndrome 'basses richesses' in sugar beet. Pest Manag. Sci..

[12] Kais B., Köhler J., Werner P., Gross J. (2023). Does the causative agent of Syndrome Basses Richesses (SBR), Candidatus Arsenophonus phytopathogenicus, alter sugar beet phloem composition or plant-emitted volatiles?. IOBC-WPRS Bulletin.

[13] Orians C.M., Jones C.G. (2001). Plants as resource mosaics: a functional model for predicting patterns of within-plant resource heterogeneity to consumers based on vascular architecture and local environmental variability. Oikos.

[14] Lucas W.J., Groover A., Lichtenberger R., Furuta K., Yadav S.-R., Helariutta Y. (2013). The plant vascular system: evolution, development and functions. J. Integr. Plant Biol..

[15] Artschwager E. (1926). Anatomy of the vegetative organs of the sugar beet. J. Agricultural Res..

[16] Musetti R., Buxa S.V., Marco F de, Loschi A., Polizzotto R., Kogel K.-H., van Bel A.J.E. (2013). Phytoplasma-triggered Ca(2+) influx is involved in sieve-tube blockage. Mol. Plant Microbe Interact..

[17] Therhaag E., Schneider B., Zikeli K., Maixner M., Gross J. (2024). Pentastiridius leporinus (linnaeus, 1761) as a vector of phloem-restricted pathogens on potatoes: 'candidatus Arsenophonus phytopathogenicus' and 'candidatus phytoplasma solani'. Insects.

[18] Eini O., Pfitzer R., Varrelmann M. (2024). Rapid and specific detection of Pentastiridius leporinus by recombinase polymerase amplification assay. Bull. Entomol. Res..

[19] Pfitzer R., Varrelmann M., Hesse G., Eini O. (2022). Molecular detection of Pentastiridius leporinus, the main vector of the syndrome 'basses richesses' in sugar beet. Insects.

[20] Behrmann S.C., Witczak N., Lang C., Schieler M., Dettweiler A., Kleinhenz B. (2022). Biology and rearing of an emerging sugar beet pest: the planthopper Pentastiridius leporinus. Insects.

[21] Simko I., Jimenez-Berni J.A., Sirault X.R.R. (2017). Phenomic approaches and tools for phytopathologists. Phytopathology.

[22] Barreto A., Paulus S., Varrelmann M., Mahlein A.-K. (2020). Hyperspectral imaging of symptoms induced by Rhizoctonia solani in sugar beet: comparison of input data and different machine learning algorithms. J. Plant Dis. Prot..

[23] Arens N., Backhaus A., Döll S., Fischer S., Seiffert U., Mock H.-P. (2016). Non-invasive presymptomatic detection of Cercospora beticola infection and identification of early metabolic responses in sugar beet. Front. Plant Sci..

[24] Mahlein A.-K., Rumpf T., Welke P., Dehne H.-W., Plümer L., Steiner U., Oerke E.-C. (2013). Development of spectral indices for detecting and identifying plant diseases. Rem. Sens. Environ..

[25] Mahlein A.-K., Steiner U., Hillnhütter C., Dehne H.-W., Oerke E.-C. (2012). Hyperspectral imaging for small-scale analysis of symptoms caused by different sugar beet diseases. Plant Methods.

[26] Hillnhütter C., Mahlein A.-K., Sikora R.A., Oerke E.-C. (2012). Use of imaging spectroscopy to discriminate symptoms caused by Heterodera schachtii and Rhizoctonia solani on sugar beet. Precis. Agric..

[27] Jahnke S., Menzel M.I., van Dusschoten D., Roeb G.W., Bühler J., Minwuyelet S. (2009). Combined MRI-PET dissects dynamic changes in plant structures and functions. Plant J..

[28] Hillnhütter C., Sikora R.A., Oerke E.-C., van Dusschoten D. (2012). Nuclear magnetic resonance: a tool for imaging belowground damage caused by Heterodera schachtii and Rhizoctonia solani on sugar beet. J. Exp. Bot..

[29] Metzner R., van Dusschoten D., Bühler J., Schurr U., Jahnke S. (2014). Belowground plant development measured with magnetic resonance imaging (MRI): exploiting the potential for non-invasive trait quantification using sugar beet as a proxy. Front. Plant Sci..

[30] Schmittgen S., Metzner R., van Dusschoten D., Jansen M., Fiorani F., Jahnke S. (2015). Magnetic resonance imaging of sugar beet taproots in soil reveals growth reduction and morphological changes during foliar Cercospora beticola infestation. J. Exp. Bot..

[31] van Dusschoten D., Metzner R., Kochs J., Postma J.A., Pflugfelder D., Bühler J. (2016). Quantitative 3D analysis of plant roots growing in soil using magnetic resonance imaging. Plant Physiol..

[32] Metzner R., Chlubek A., Bühler J., Pflugfelder D., Schurr U., Huber G. (2022). In vivo imaging and quantification of carbon tracer dynamics in nodulated root systems of pea plants. Plants.

[33] Hinz C., Jahnke S., Metzner R., Pflugfelder D., Scheins J.J., Streun M., Koller R. (2024). Setup and characterisation according to NEMA NU 4 of thephenoPET scanner, a PET system dedicated for plant sciences. Phys. Med. Biol..

[34] Karve A.A., Alexoff D., Kim D., Schueller M.J., Ferrieri R.A., Babst B.A. (2015). In vivo quantitative imaging of photoassimilate transport dynamics and allocation in large plants using a commercial positron emission tomography (PET) scanner. BMC Plant Biol..

[35] Miyoshi Y., Soma F., Yin Y.-G., Suzui N., Noda Y., Enomoto K. (2022). Rice immediately adapts the dynamics of photosynthates translocation to roots in response to changes in soil water environment. Front. Plant Sci..

[36] Yu P., Li C., Li M., He X., Wang D., Li H. (2024). Seedling root system adaptation to water availability during maize domestication and global expansion. Nat. Genet..

[37] Pflugfelder D., Metzner R., van Dusschoten D., Reichel R., Jahnke S., Koller R. (2017). Non-invasive imaging of plant roots in different soils using magnetic resonance imaging (MRI). Plant Methods.

[38] Kim D., Alexoff D.L., Schueller M., Babst B., Ferrieri R., Fowler J.S., Schlyer D.J. (2014). The design and performance of a portable handheld (11)CO2 delivery system. Appl. Radiat. Isot..

[39] Zübert C., Kube M. (2021). Application of TaqMan real-time PCR for detecting 'candidatus Arsenophonus phytopathogenicus' infection in sugar beet. Pathogens.

[40] Christensen N.M., Nicolaisen M., Hansen M., Schulz A. (2004). Distribution of phytoplasmas in infected plants as revealed by real-time PCR and bioimaging. Mol. Plant Microbe Interact..

[41] Tatineni S., Sagaram U.S., Gowda S., Robertson C.J., Dawson W.O., Iwanami T., Wang N. (2008). In planta distribution of 'candidatus liberibacter asiaticus' as revealed by polymerase chain reaction (PCR) and real-time PCR. Phytopathology.

[42] Joy K.W. (1964). Translocation in sugar beet: I. Assimilation of 14CO2, and distribution of materials from leaves. J. Exp. Bot..

[43] Greenberg J.T., Yao N. (2004). The role and regulation of programmed cell death in plant-pathogen interactions. Cell. Microbiol..

[44] Rodrigues C.M., Müdsam C., Keller I., Zierer W., Czarnecki O., Corral J.M. (2020). Vernalization alters sink and source identities and reverses phloem translocation from taproots to shoots in sugar beet. Plant Cell.

[45] Kakouridis A., Yuan M., Nuccio E.E., Hagen J.A., Fossum C.A., Moore M.L. (2024). Arbuscular mycorrhiza convey significant plant carbon to a diverse hyphosphere microbial food web and mineral-associated organic matter. New Phytol..

[46] Märländer B., Hoffmann C., Koch H.-J., Ladewig E., Merkes R., Petersen J., Stockfisch N. (2003). Environmental situation and yield performance of the sugar beet crop in Germany: heading for sustainable development. J. Agron. Crop Sci..

[47] Hoffmann C.M. (2010). Sucrose accumulation in sugar beet under drought stress. J. Agron. Crop Sci..

[48] Rossi V., Meriggi P., Biancardi E., Rosso F., Asher M.J.C., Holtschulte B., Molard M.R., Rosso F., Steinruecken G., Beckers R. (2000).

[49] Nause N., Ispizua Yamati F.R., Seidel M., Mahlein A.-K., Hoffmann C.M. (2023). Workflow for phenotyping sugar beet roots by automated evaluation of cell characteristics and tissue arrangement using digital image processing. Plant Methods.

[50] Kleuker G., Hoffmann C.M. (2021). Tissue strength of sugar beet root genotypic variation and environmental impact. Crop Sci..

[51] Braun D.M., Wang L., Ruan Y.-L. (2014). Understanding and manipulating sucrose phloem loading, unloading, metabolism, and signalling to enhance crop yield and food security. J. Exp. Bot..

[52] Chen L.-Q., Hou B.-H., Lalonde S., Takanaga H., Hartung M.L., Qu X.-Q. (2010). Sugar transporters for intercellular exchange and nutrition of pathogens. Nature.

[53] Reyer A., Bazihizina N., Jaślan J., Scherzer S., Schäfer N., Jaślan D. (2024). Sugar beet PMT5a and STP13 carriers suitable for proton-driven plasma membrane sucrose and glucose import in taproots. Plant J..

