# Supplementary material for: Syndrome “basses richesses” disease induced structural deformations and sectorial distribution of photoassimilates in sugar beet taproot revealed by combined MRI-PET imaging
Source: Plant Phenomics. 2025 May 15;7(2):100053. doi: 10.1016/j.plaphe.2025.100053 (PMC12709939; doi:10.1016/j.plaphe.2025.100053)
Supplement: Multimedia component 1 [file mmc1.pdf]

Article title: Syndrome “basses richesses” disease induced structural deformations and sectorial distribution of photoassimilates in sugar beet taproot revealed by combined MRI-PET

Authors: Kwabena Agyei, Justus Detring, Ralf Metzner, Gregor Huber, Daniel Pflugfelder, Omid Eini, Mark Varrelmann, Anne-Katrin Mahlein and Robert Koller

Supplementary Data

Fig. S1 Approximate positions for taproot cross-sectioning.

Fig. S2-S12 Cross-sectional anatomical features of control taproot samples C2-C12.

Fig. S13-S19 Cross-sectional anatomical features of diseased taproot samples D2-D8.

Fig. S20-S22 Tracer distribution within taproots for control samples C2-C4.

Fig. S23 Tracer distribution within taproots for diseased sample D2.

Table S1 Primers and probe for detection of ‘*Ca.* A. phytopathogenicus’.

Table S2 Mean Cq values of qPCR.

Table S3 Taproot diameter and fresh weight after harvest.

Table S4 Heterogeneity of tracer distribution within taproots.

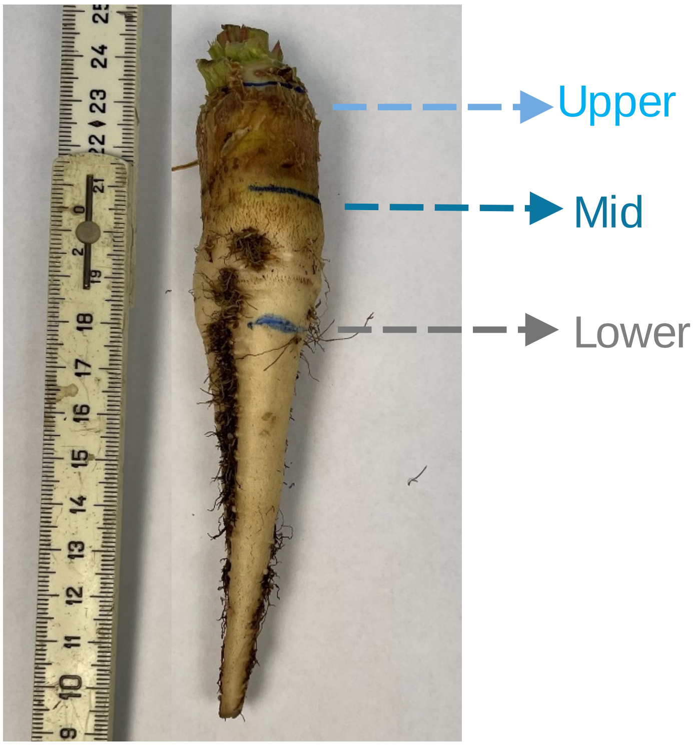

Fig. S1. Approximate positions for cross-sectional slicing, RGB imaging and sample taking for qPCR during destructive analysis

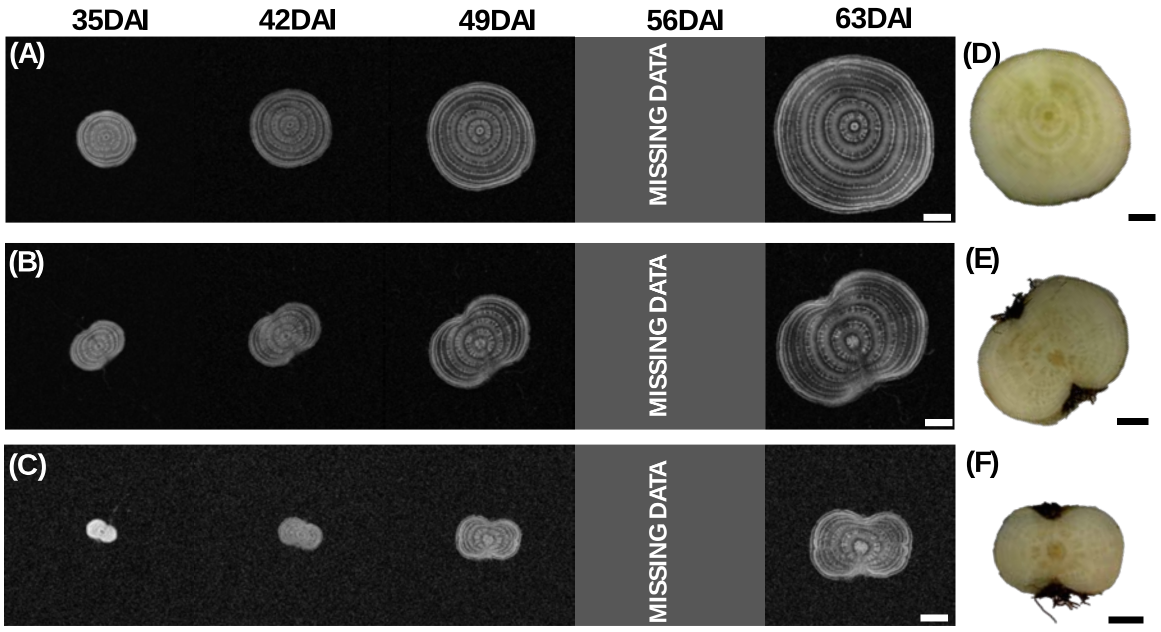

Fig. S2. Cross-sectional anatomical features of control sample C2. (A-C) MRI and (D-F) RBG images for upper (A,D), middle (B,E) and lower (C,F) taproot slices. Scale bar, 0.5 cm.

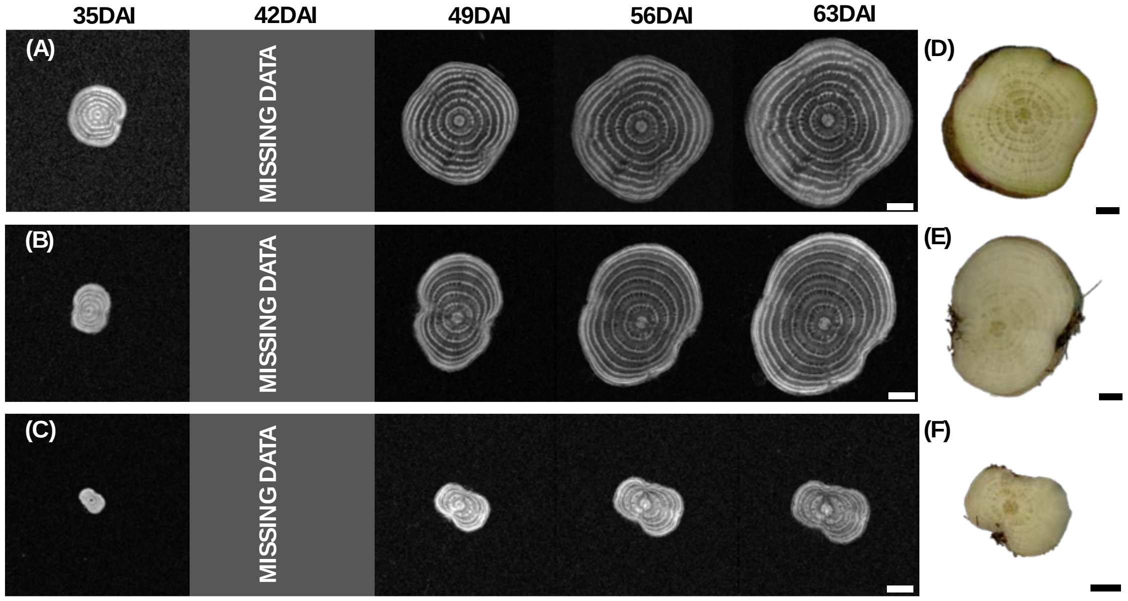

Fig. S3. Cross-sectional anatomical features of control sample C3. (A-C) MRI and (D-F) RBG images for upper (A,D), middle (B,E) and lower (C,F) taproot slices. Scale bar, 0.5 cm.

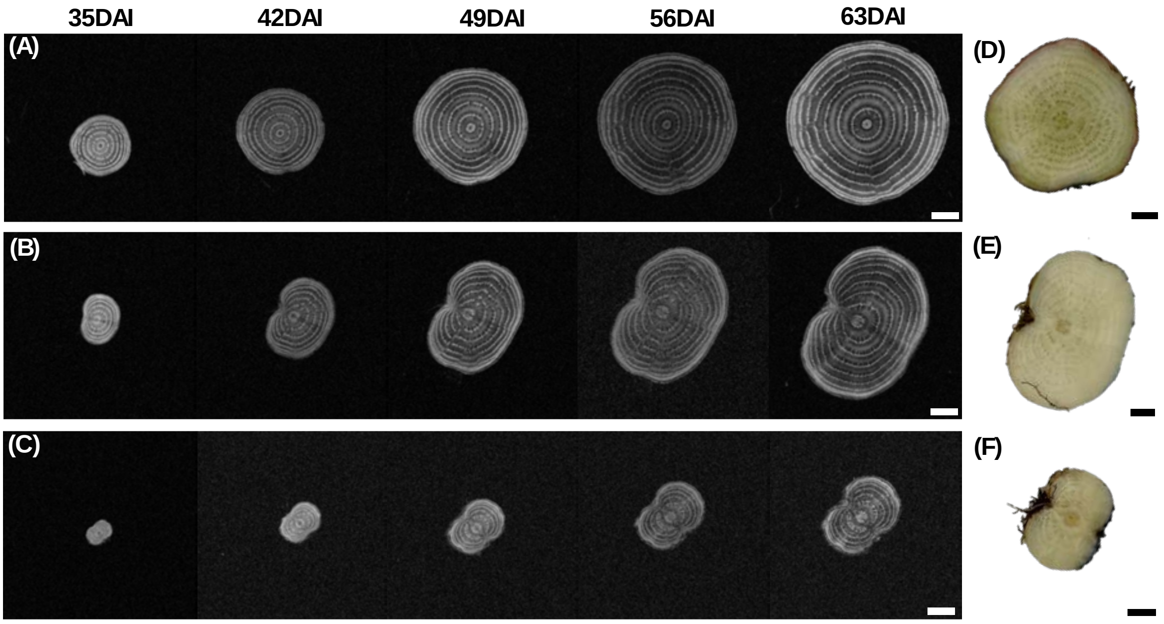

Fig. S4. Cross-sectional anatomical features of control sample C4. (A-C) MRI and (D-F) RBG images for upper (A,D), middle (B,E) and lower (C,F) taproot slices. Scale bar, 0.5 cm.

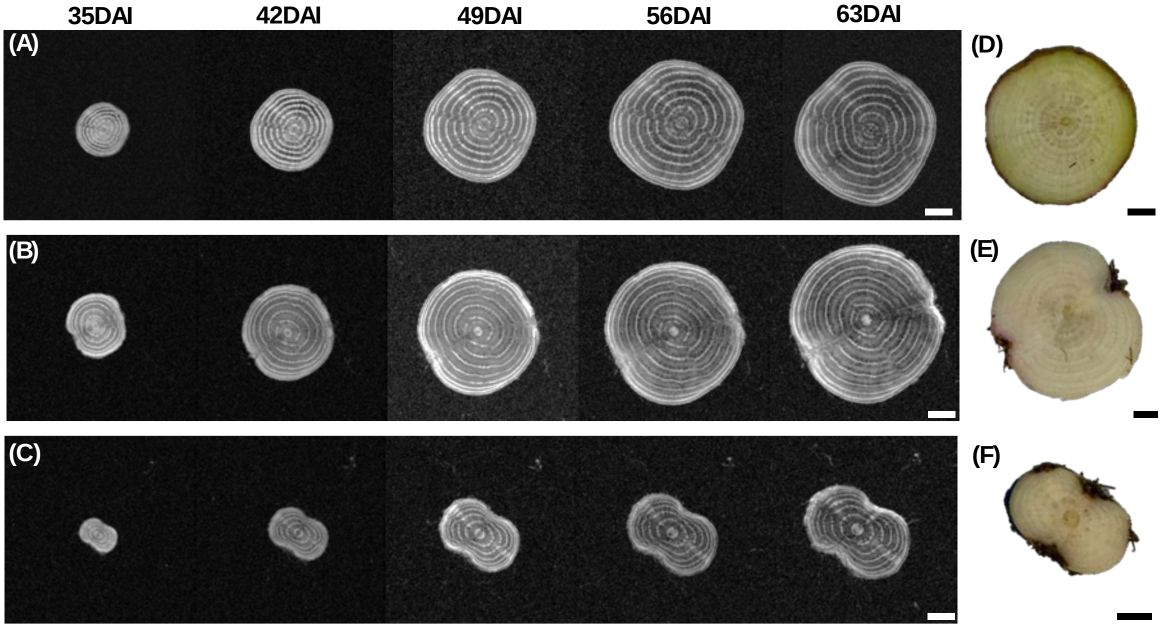

Fig. S5. Cross-sectional anatomical features of control sample C5. (A-C) MRI and (D-F) RBG images for upper (A,D), middle (B,E) and lower (C,F) taproot slices. Scale bar, 0.5 cm.

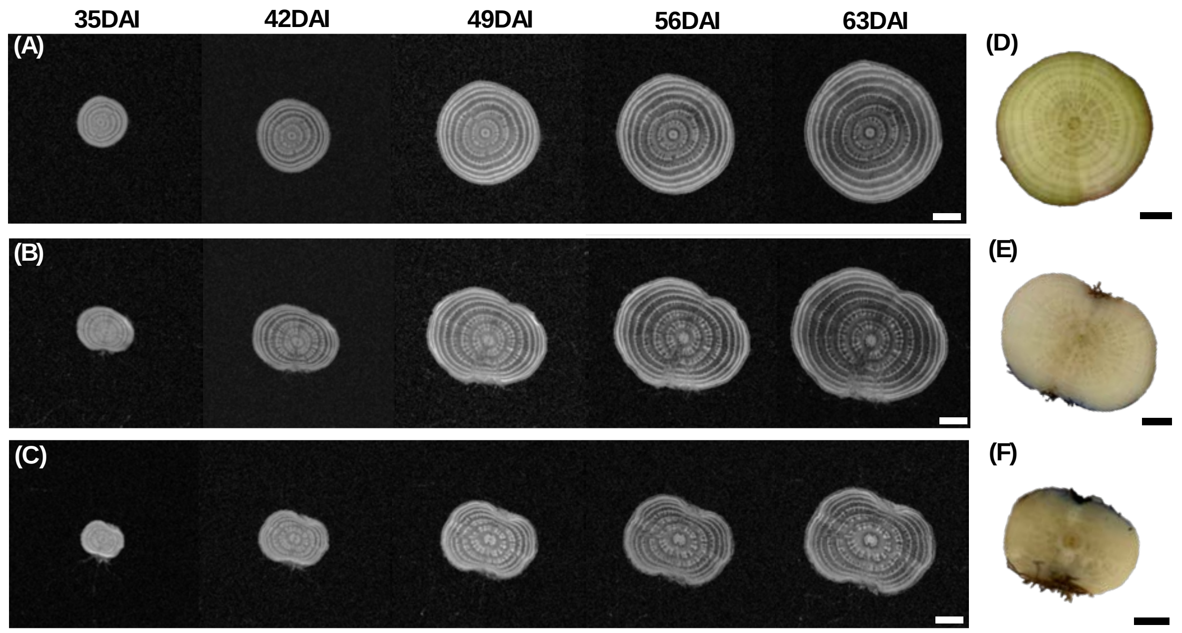

Fig. S6. Cross-sectional anatomical features of control sample C6. (A-C) MRI and (D-F) RBG images for upper (A,D), middle (B,E) and lower (C,F) taproot slices. Scale bar, 0.5 cm.

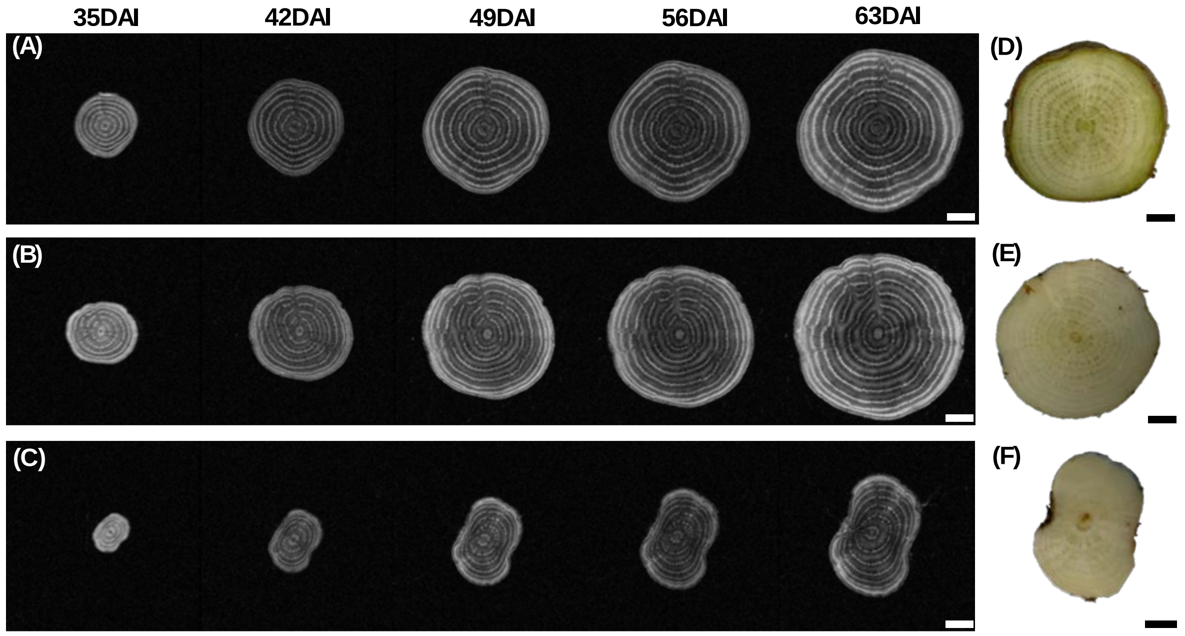

Fig. S7. Cross-sectional anatomical features of control sample C7. (A-C) MRI and (D-F) RBG images for upper (A,D), middle (B,E) and lower (C,F) taproot slices. Scale bar, 0.5 cm.

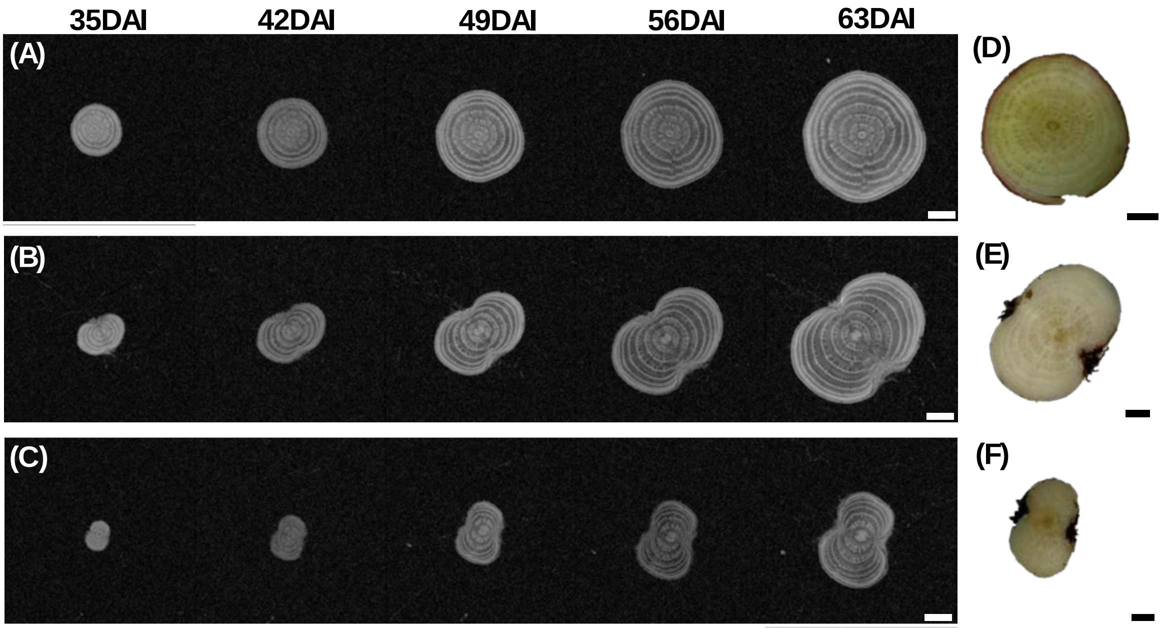

Fig. S8. Cross-sectional anatomical features of control sample C8. (A-C) MRI and (D-F) RBG images for upper (A,D), middle (B,E) and lower (C,F) taproot slices. Scale bar, 0.5 cm.

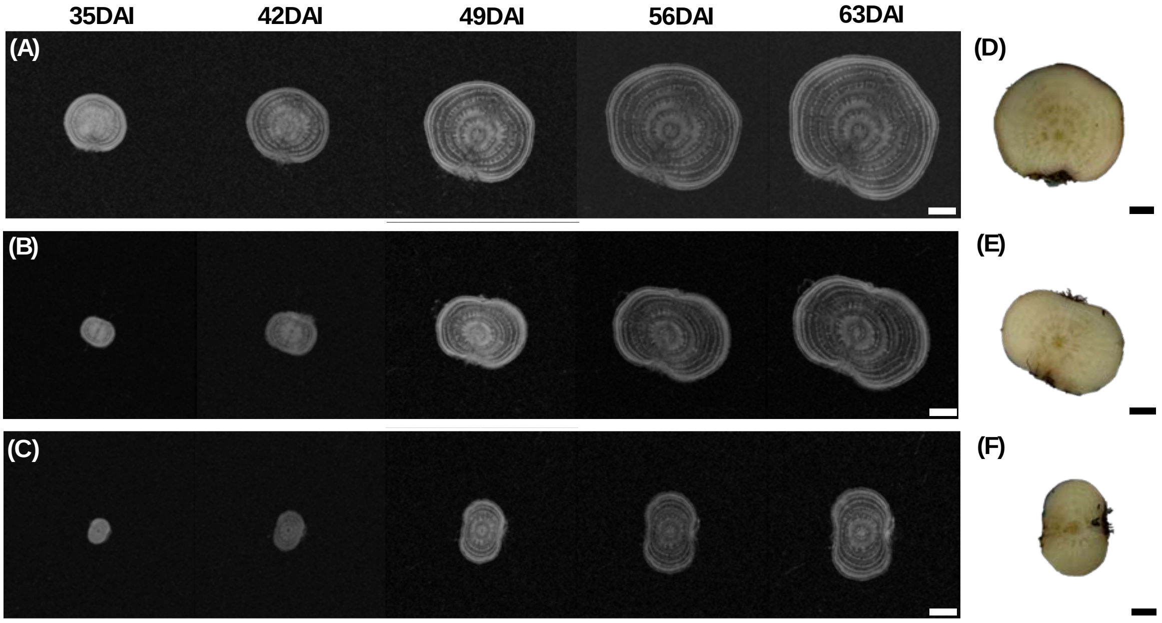

Fig. S9. Cross-sectional anatomical features of control sample C9. (A-C) MRI and (D-F) RBG images for upper (A,D), middle (B,E) and lower (C,F) taproot slices. Scale bar, 0.5 cm.

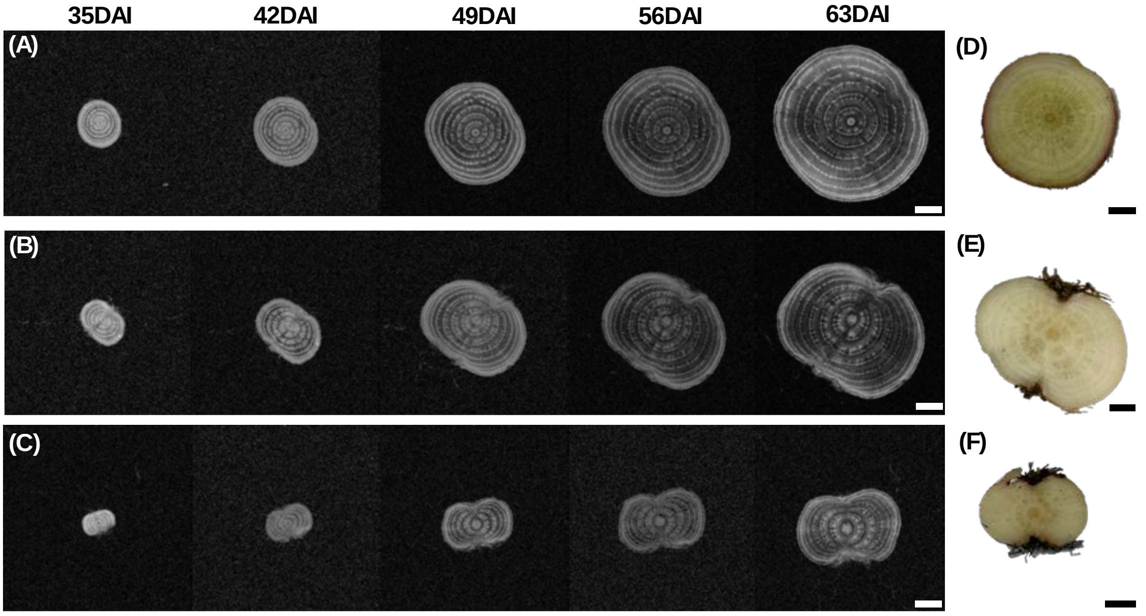

Fig. S10. Cross-sectional anatomical features of control sample C10. (A-C) MRI and (D-F) RBG images for upper (A,D), middle (B,E) and lower (C,F) taproot slices. Scale bar, 0.5 cm.

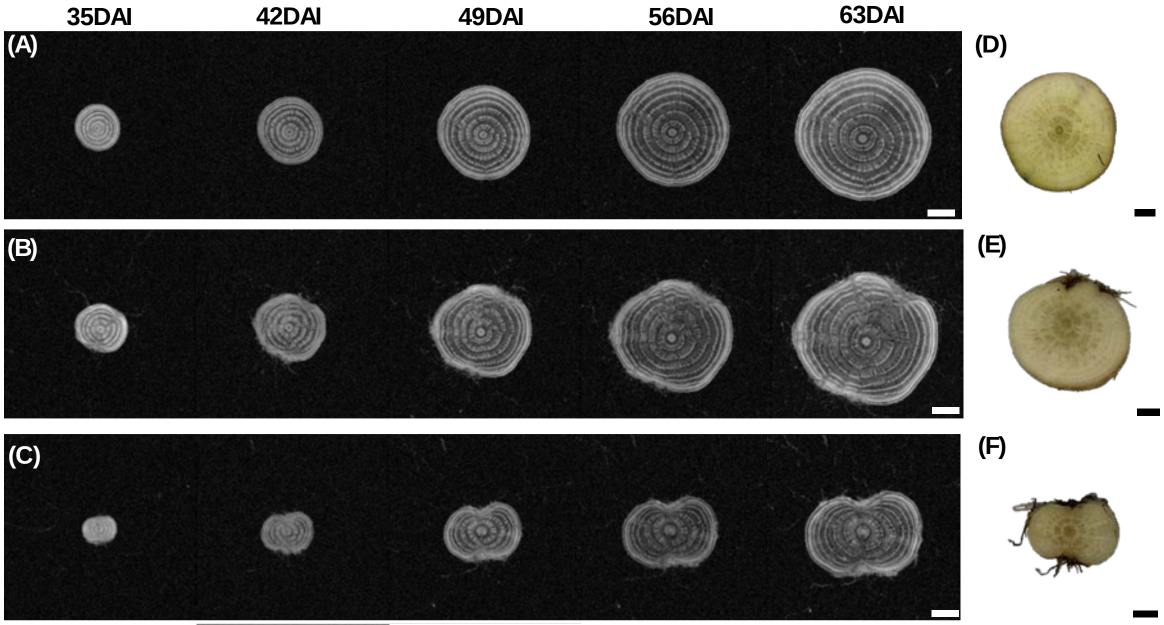

Fig. S11. Cross-sectional anatomical features of control sample C11. (A-C) MRI and (D-F) RBG images for upper (A,D), middle (B,E) and lower (C,F) taproot slices. Scale bar, 0.5 cm.

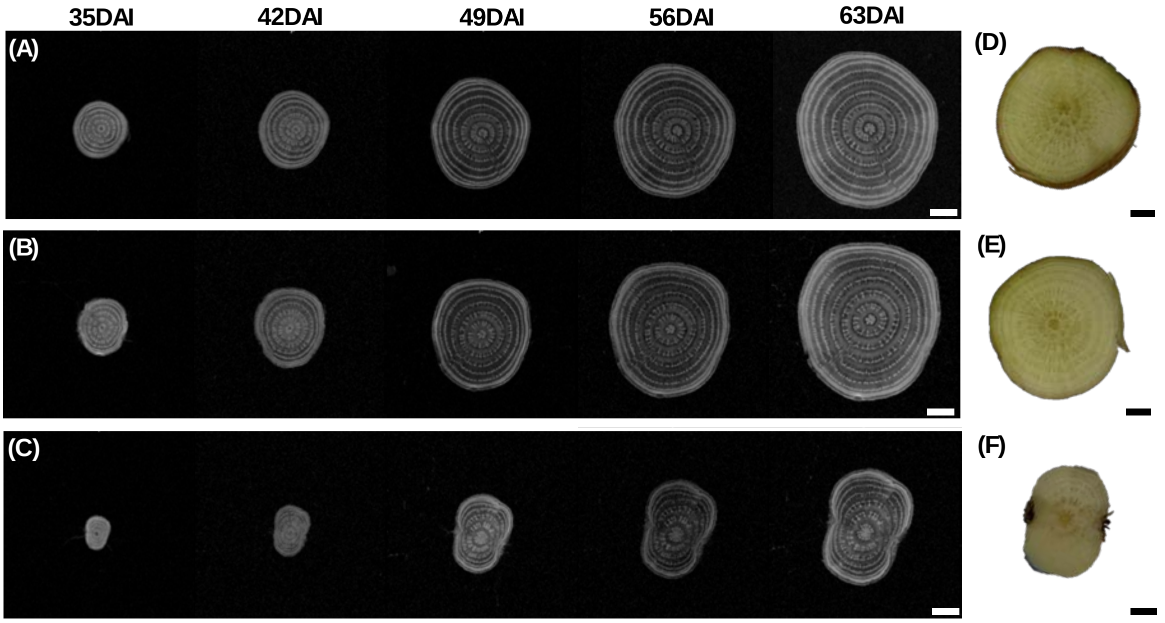

Fig. S12. Cross-sectional anatomical features of control sample C12. (A-C) MRI and (D-F) RBG images for upper (A,D), middle (B,E) and lower (C,F) taproot slices. Scale bar, 0.5 cm.

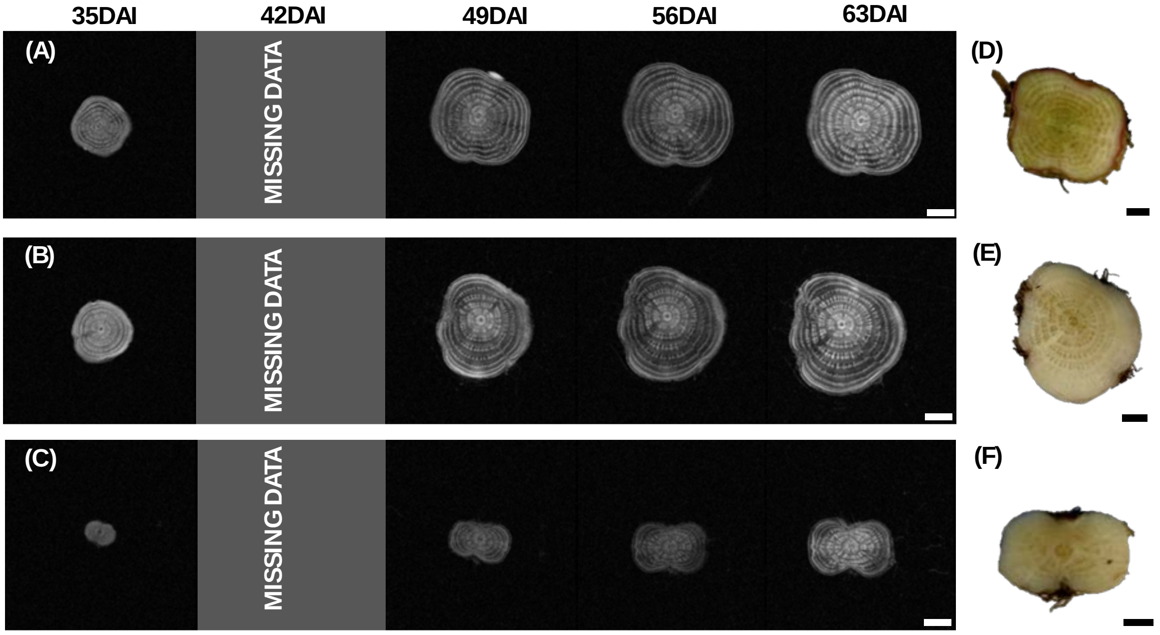

Fig. S13. Cross-sectional anatomical features of diseased sample D2. (A-C) MRI and (D-F) RBG images for upper (A,D), middle (B,E) and lower (C,F) taproot slices. Scale bar, 0.5 cm.

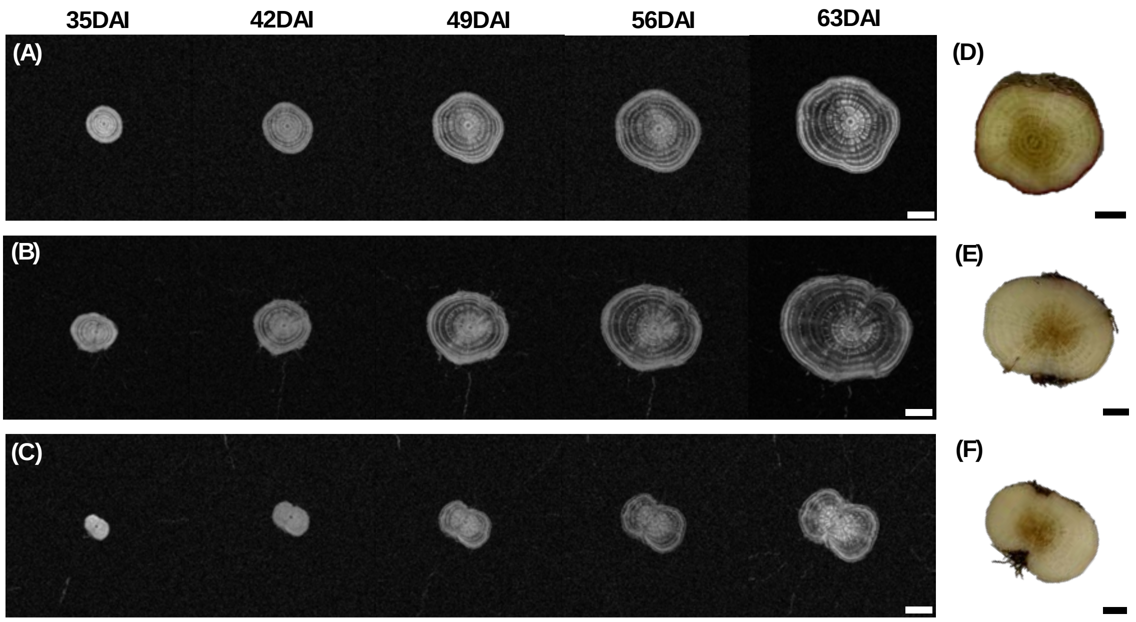

Fig. S14. Cross-sectional anatomical features of diseased sample D3. (A-C) MRI and (D-F) RBG images for upper (A,D), middle (B,E) and lower (C,F) taproot slices. Scale bar, 0.5 cm.

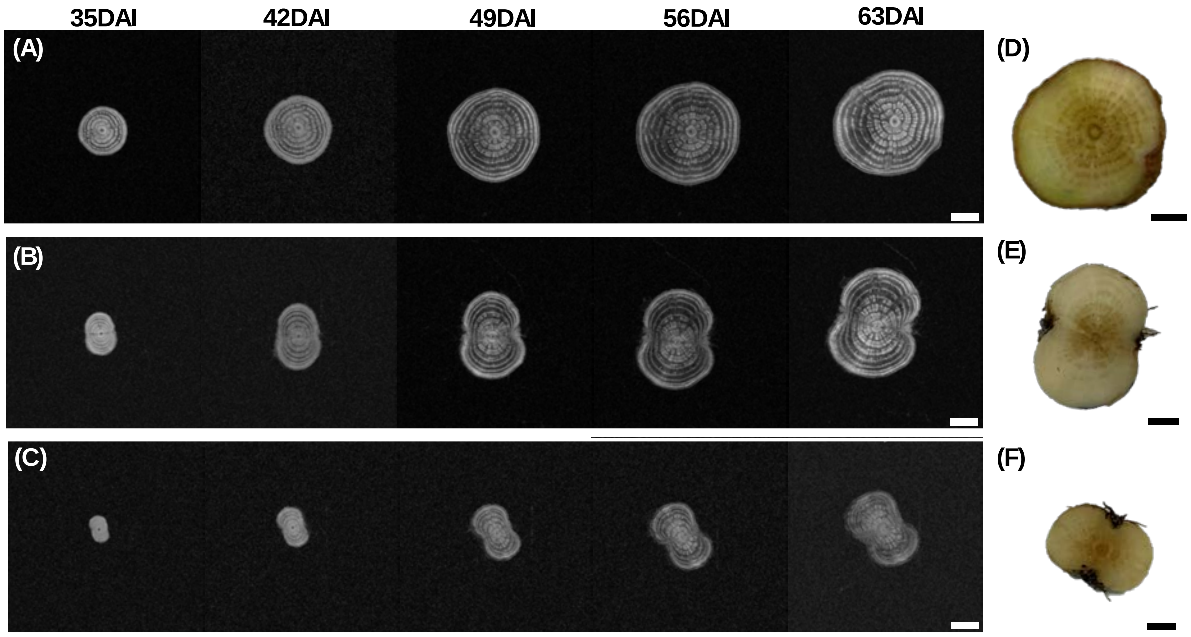

Fig. S15. Cross-sectional anatomical features of diseased sample D4. (A-C) MRI and (D-F) RBG images for upper (A,D), middle (B,E) and lower (C,F) taproot slices. Scale bar, 0.5 cm.

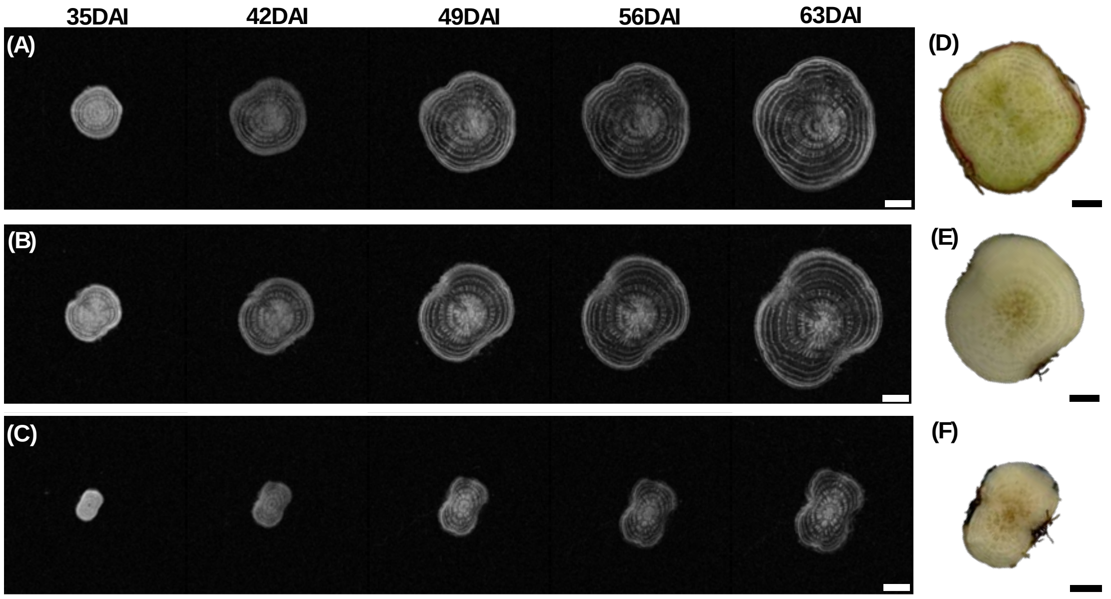

Fig. S16. Cross-sectional anatomical features of diseased sample D5. (A-C) MRI and (D-F) RBG images for upper (A,D), middle (B,E) and lower (C,F) taproot slices. Scale bar, 0.5 cm.

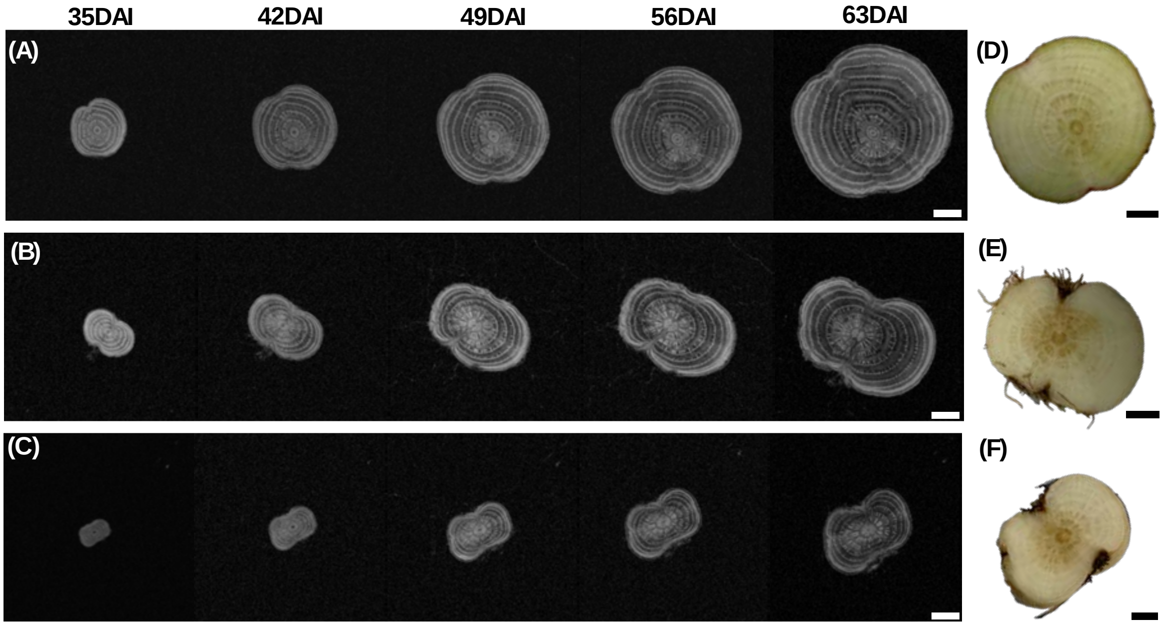

Fig. S17. Cross-sectional anatomical features of diseased sample D6. (A-C) MRI and (D-F) RBG images for upper (A,D), middle (B,E) and lower (C,F) taproot slices. Scale bar, 0.5 cm.

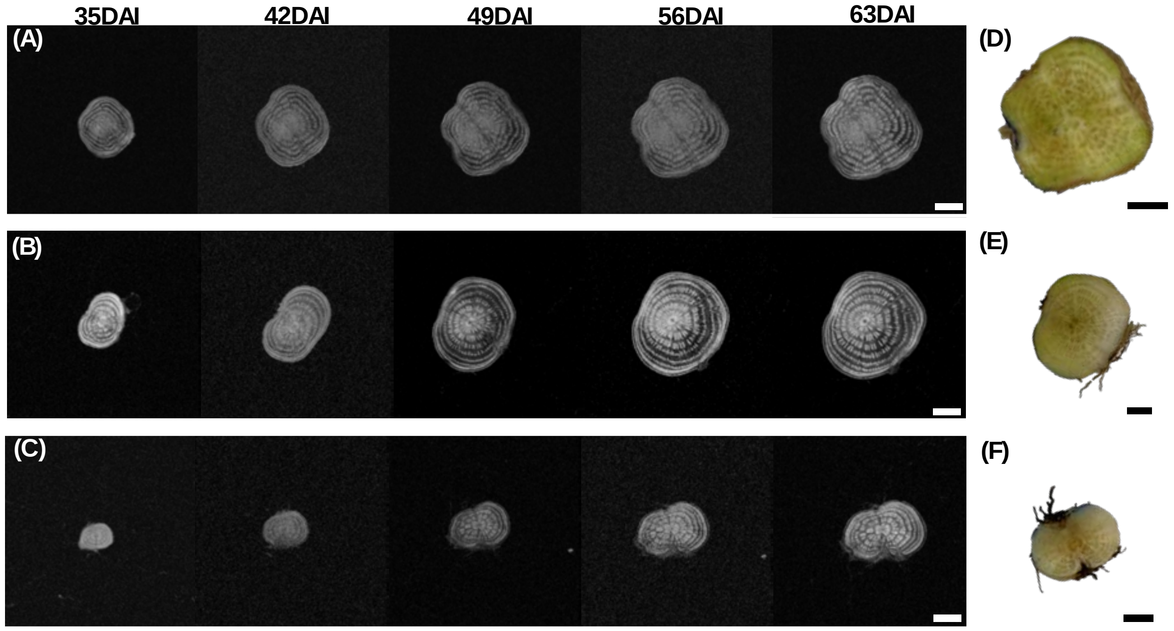

Fig. S18. Cross-sectional anatomical features of diseased sample D7. (A-C) MRI and (D-F) RBG images for upper (A,D), middle (B,E) and lower (C,F) taproot slices. Scale bar, 0.5 cm.

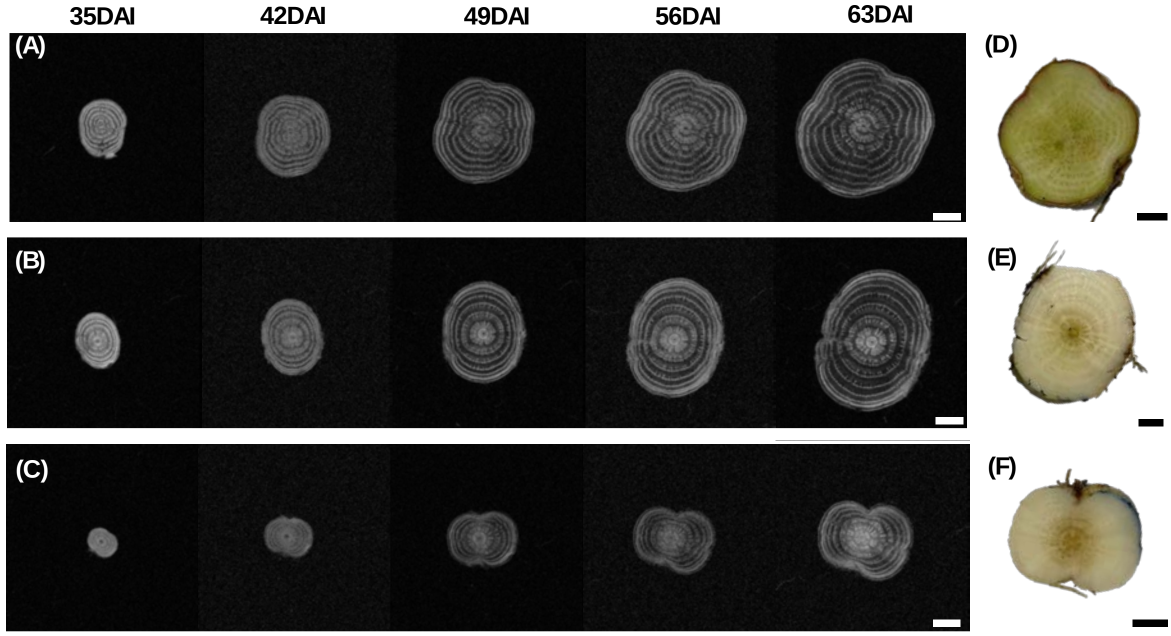

Fig. S19. Cross-sectional anatomical features of diseased sample D8. (A-C) MRI and (D-F) RBG images for upper (A,D), middle (B,E) and lower (C,F) taproot slices. Scale bar, 0.5 cm.

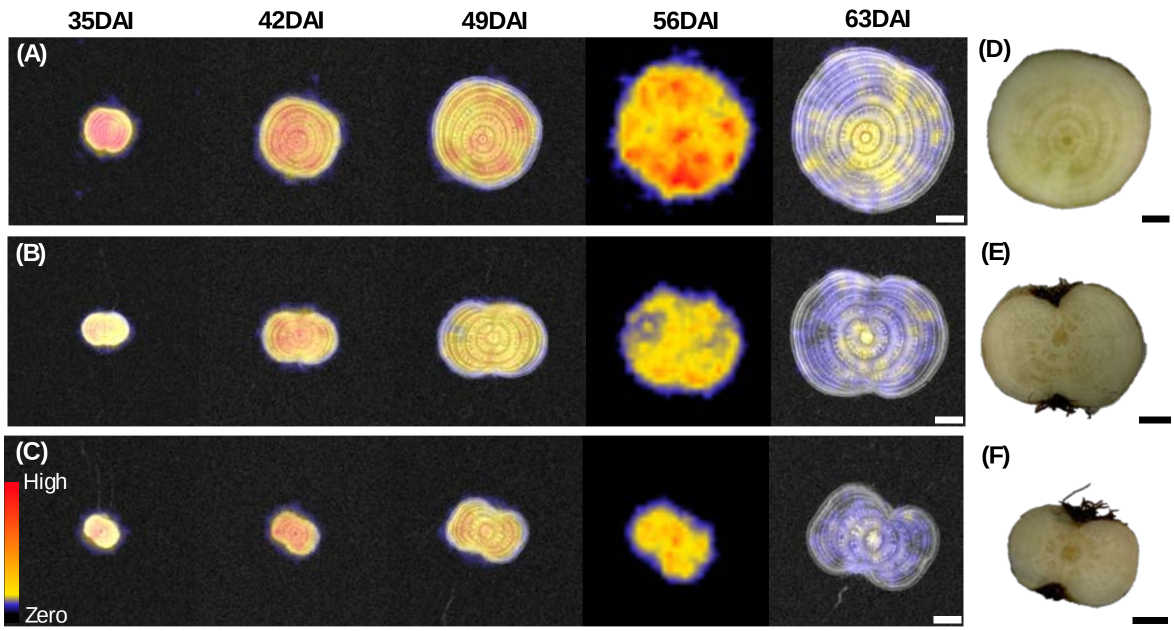

Fig. S20. MRI-PET coregistration indicating tracer distribution within taproot for control sample C2. MRI data is missing for 56 DAI. Scale bar, 0.5 cm.

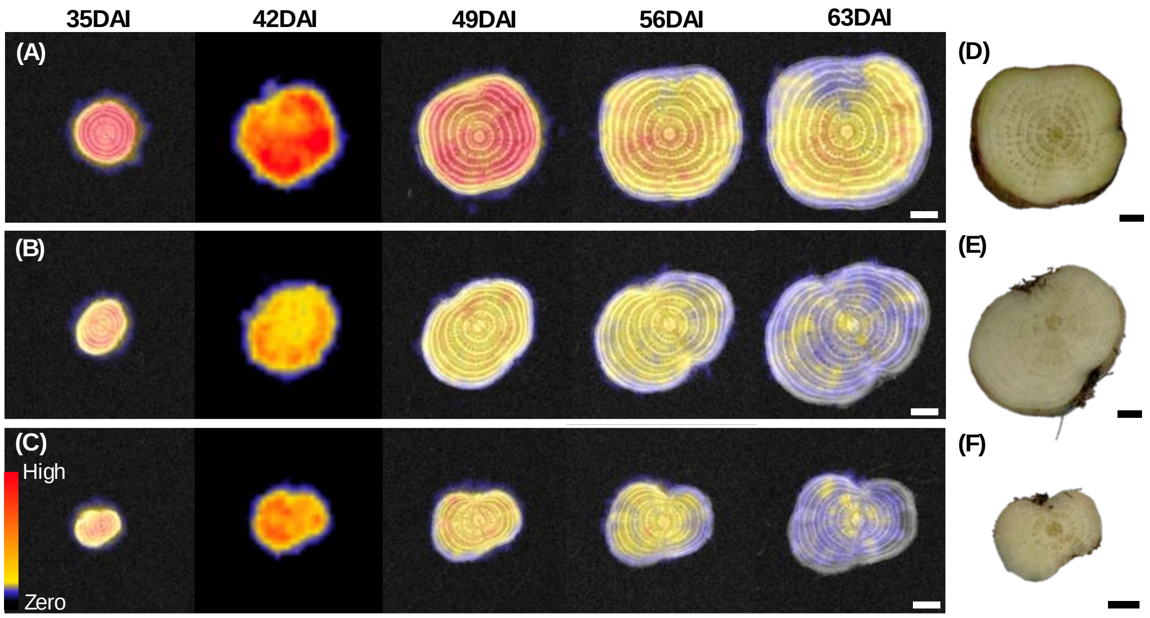

Fig. S21. MRI-PET coregistration indicating tracer distribution within taproot for control sample C3. MRI data is missing for 42 DAI. Scale bar, 0.5 cm.

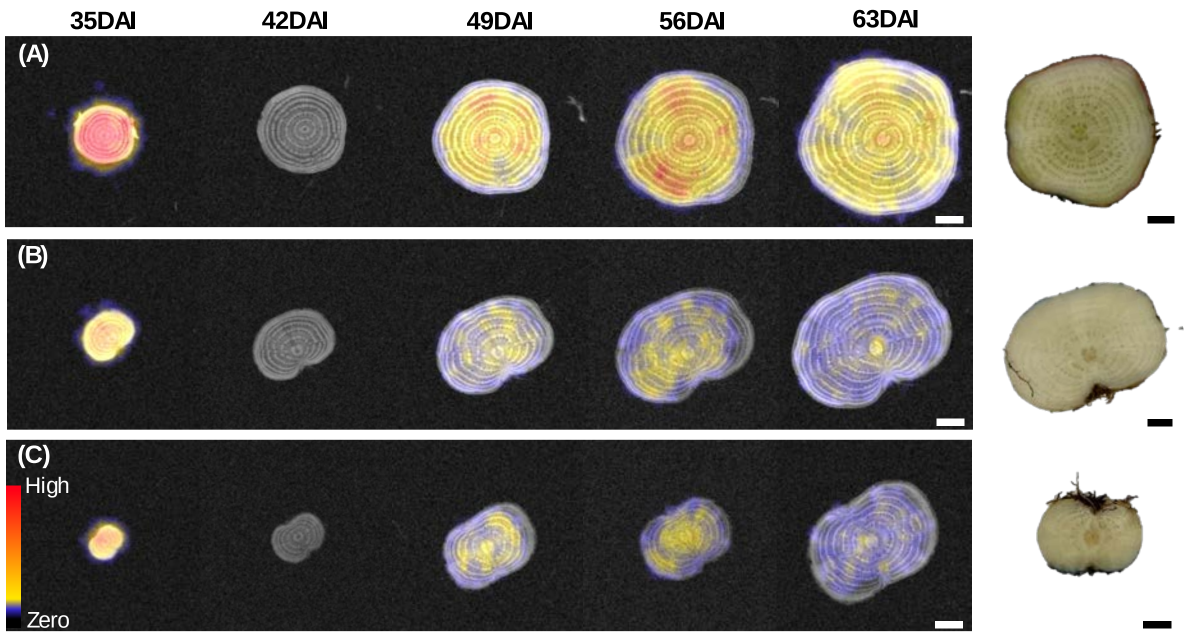

Fig. S22 MRI-PET coregistration indicating tracer distribution within taproot for control sample C4. PET data is missing for 42 DAI. Scale bar, 0.5 cm.

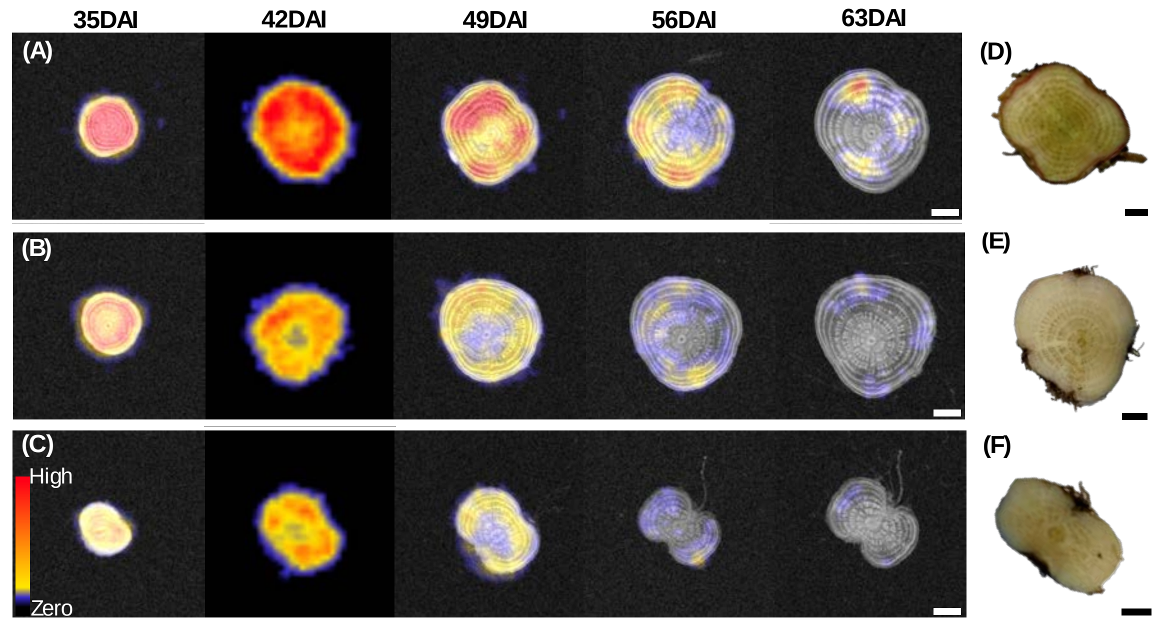

Fig. S23 MRI-PET coregistration indicating tracer distribution within taproot for diseased sample D2. MRI data is missing for 42 DAI. Scale bar, 0.5 cm.

Table S1 Primers and probe for detection of '*Ca* A. phytopatogenicus'**.**

	Sequence (5'-3')	Tm	labelling	Product size
Primer 1	tggaactcacagtagcggtt	58		90 nt
Primer 2	cacttttgccgctgatagtca	57		
Probe 1	aactcctgttgtttataaccagg	55	6-FAM/BHQ-1	

Table S2 Mean quantification cycle (Cq) values for each sample after qPCR for upper, mid and lower beet sections for each analyzed sample**.**

Sample ID	Upper	Mid	Lower
C3	N/D	N/D	N/D
C4	N/D	N/D	N/D
C10	N/D	N/D	N/A
C12	N/D	N/D	N/D
D1	27.01	25.87	24.91
D2	26.24	25.85	24.48
D3	26.44	28.40	26.42
D4	25.13	26.03	23.64
D5	29.70	28.50	28.53
D6	28.11	27.01	25.41
D7	25.93	26.03	24.34
D8	26.52	26.86	25.34

N/D, not detected, i.e. Cq > 35; N/A, not available.

Table S3 Taproot fresh weight and diameter after harvest.

Sample ID	Fresh weight [g]	Diameter [mm]
C1	43.8	26.6
C2	48.7	30.3
C3	52.4	32.7
C4	43.6	29.4
C5	44.5	31.5
C6	43.6	29.1
C7	48	30.2
C8	37.3	27
C9	35.5	30.5
C10	42.29	29.2
C11	40.7	27.7
C12	42.5	30.6
D1	28.2	24.2
D2	31.8	26.7
D3	30.3	24.4
D4	37.4	26
D5	30.5	25.3
D6	41.7	29.5
D7	27.4	22.1
D8	41.7	27.4

Table S4 Heterogeneity (*H*) in tracer distribution within several slices for each measured beet.

	Control				Infected	
DAI	C1	C2	C3	C4	D1	D2
35	0.36 ± 0.07	0.54 ± 0.07	0.34 ± 0.11	0.72 ± 0.34	0.64 ± 0.16	0.32 ± 0.06
42	0.45 ± 0.06	0.84 ± 0.25	N/A	N/A	0.78 ± 0.18	N/A
49	0.57 ± 0.07	0.59 ± 0.08	0.30 ± 0.04	0.64 ± 0.05	1.0 ±0.13	0.66 ±0.03
56	0.71 ± 0.17	N/A	0.42 ± 0.03	0.72 ± 0.05	1.06 ±0.26	1.08 ±0.11
63	0.44 ± 0.03	0.71 ± 0.09	0.50 ± 0.15	0.51 ± 0.06	1.33 ±0.17	1.40 ±0.14

DAI, days after inoculation. Values are mean ± STD: n= 10. N/A indicates missing data due to unsuccessful measurements.
